# Synaptic transmission: Munc13 assembles onto PI(4,5)P_2_-rich domains into trimers that cooperate to capture vesicles

**DOI:** 10.1073/pnas.2523347123

**Published:** 2026-02-11

**Authors:** Feng Li, Abhijith Radhakrishnan, Sudhanshu Gautam, Gabriel Diaz, Ramalingam Venkat Kalyana Sundaram, Jeff Coleman, Hong Zheng, Kirill Grushin, Matthieu Chavent, James E. Rothman, Frederic Pincet

**Affiliations:** ^a^Department of Cell Biology, School of Medicine, Yale University, New Haven, CT 06520; ^b^Nanobiology Institute, Yale University West Campus, West Haven, CT 06516; ^c^Laboratoire de Microbiologie et Génétique Moléculaires, Centre de Biologie Intégrative, Université de Toulouse, CNRS, Université Paul Sabatier, Toulouse F-31400, France; ^d^Laboratoire de Physique de l’Ecole normale supérieure, Ecole Normale Supérieure, Université Paris Sciences Lettres, CNRS, Sorbonne Université, Université Paris-Cité, Paris F-75005, France

**Keywords:** C2 domains, Syntaxin-1A, diacylglycerol, TIRF, cryoelectron tomography

## Abstract

Munc13-1 is a key protein involved in priming synaptic vesicles for rapid release at the presynaptic plasma membrane. It was previously revealed that Munc13-1 cycles between at least two alternate conformations, an upright (open) molecular conformation organized as a trimer and a lateral (closed) conformation organized as a hexagon. Munc13 binds PI(4,5)P_2_, a plasma membrane phospholipid essential for vesicle docking and fusion. We report that Munc13 is recruited to PI(4,5)P_2_ domains induced by a Syntaxin-1A juxta-membrane peptide in supported bilayers. Statistical analysis of the copy numbers of Munc13 within the domains suggests that Munc13 exists in clusters of three molecules, i.e., it assembles into trimers. The trimeric clusters disappear with engineered interface mutations disrupting the upright trimers in cryo-EM as well as reconstituted vesicle priming suggesting the trimers observed on bilayers are identical to the upright trimer structures. These upright trimers can also be identified by cryo-electron tomography on vesicles containing PI(4,5)P_2_ and Syntaxin-1A. Clusters of 3 or more Munc13 trimers forming on PI(4,5)P_2_ domains efficiently capture phosphatidylserine-containing small unilamellar vesicles via their C_2_C domains, as shown by the effect of mutations that disrupt synaptic vesicle binding in synapses. We propose a two-step model for vesicle priming: i) synaptic vesicles are captured by clusters of upright trimers of Munc13 that self-assemble within PI(4,5)P_2_ enriched domains; ii) these trimers transition into lateral hexamers when vesicles have bound and the transition would be promoted when the closed conformation of Munc13 is stabilized by diacylglycerol binding, resulting from Ca^2+^-dependent hydrolysis of PI(4,5)P_2_.

## Significance

Munc13-1 primes synaptic vesicles for rapid release at the presynaptic membrane. Reconstitution on supported bilayers and Cryo-EM reveal that Munc13-1 cycles between upright (trimeric) and lateral (hexameric) conformations. Munc13 binds PI(4,5)P_2_, a key lipid for vesicle docking and fusion. We show that Munc13 assembles into trimer clusters on PI(4,5)P_2_ domains. These clusters efficiently capture vesicles via C_2_C domains. Trimer interface mutations disrupt vesicle priming. These observations prompted us to propose that priming begins with vesicle capture by Munc13 trimers, which transition into lateral hexamers upon vesicle binding, a process promoted by diacylglycerol production from PI(4,5)P_2_ hydrolysis.

At the synapse, the active zone is a highly dynamic region of the presynaptic plasma membrane where synaptic vesicles line up to release their encapsulated neurotransmitters upon the arrival of an action potential (1–4). Some of these vesicles (5) are primed to enable release in less than a millisecond. Following neurotransmitter release, replacement vesicles must then be rapidly primed. This cycle of “release by fusion/replenishment” can repeat several times when the synapse receives a train of sequential action potentials (6–9). When such a train is sustained long enough, all vesicles capable of quickly reaching the primed state become depleted. Artificially induced trains of action potentials are a common method to experimentally estimate the size of this readily releasable pool (RRP) of synaptic vesicles.

The specialized molecular machinery responsible for priming synaptic vesicles is well known, but how these SNARE (VAMP, Syntaxin, SNAP25), SNARE-chaperoning (Munc13, Munc18, Synaptophysin), and SNARE-clamping/Ca^2+^-sensing (Synaptotagmin, Complexin) proteins are spatially arranged and coordinate with each other to produce the energy-rich readily releasable, primed state is unclear (10–15).

Recent structural and biochemical studies of the synaptic SNAREpin-assembling chaperone Munc13-1, a 25 nm long banana-shaped protein long known to play a key role (16–21), suggest it assembles into a series of oligomers that successively ratchet the synaptic vesicle toward the presynaptic plasma membrane as it assembles two successive waves of SNAREpins (15, 22–24). In particular, cryo-EM tomography of lipid bilayer-Munc13 cocrystals uncovered two distinct membrane-bound oligomeric arrangements of Munc13 (25). One oligomer consists of an upright trimer whose Munc13 subunits extend in a fully open conformation between two apposed bilayers, separating them by ~21 nm, bound at one end (corresponding to the plasma membrane) by its PI(4,5)P_2_-binding C_2_B domain, and at the other end bound by its synaptic vesicle-binding C_2_C domain to the apposed bilayer. The second oligomer consists of six copies of Munc13 in a distinct closed conformation, which is achieved by a simple rigid body rotation from the open conformation that positions its diacylglycerol-binding C_1_ domain against the plasma membrane to enable this lipid to bind. This rotation, together with the curved shape of the central MUN domain, repositions the C_2_C domain parallel to the membrane enabling it to engage laterally with a neighboring Munc13 at a 120° angle, such that six copies form a closed ring, termed the lateral hexamer. A vesicle bound to the top of this ring would now be ~14 nm from the plasma membrane, close enough for SNAREpins to assemble (25).

These two structures likely represent key intermediates in the docking and priming of the fusion machinery during synaptic vesicle transmission, as evidenced by the elimination of synaptic transmission by mutations that disrupt the trimer and hexamer interfaces in the worm *Caenorhabditis elegans* and in reconstituted vesicle priming (15). The greater vesicle–membrane separation observed for upright trimers compared with lateral hexamers suggests that the upright trimer precedes the lateral hexamer conformation. In this scenario, the upright trimer would constitute a transient intermediate en route to synaptic vesicle priming. A key prediction of our model for priming (25) is that each oligomer should be capable of separate self-assembly outside of the crystal in which they were discovered. This has already been demonstrated for lateral hexamers, which can assemble into hexameric clusters on diacylglycerol (DAG)-rich domains in supported bilayers as shown by single molecule counting methods (22, 26). The clusters on bilayers correspond to lateral hexamers because they are disrupted by unique interface mutations, and as predicted are stabilized by DAG (25). Each such hexamer can capture a single phosphatidylserine-containing vesicle, a model of a synaptic vesicle (22).

In this paper, we report studies of the second oligomeric state of Munc13 when it is bound to bilayers lacking DAG but still containing PI(4,5)P_2_. As predicted by our model for priming, the main oligomer is now a trimer, and we did not observe any hexamer, confirming their requirement for DAG. Efficient trimer assembly required concentration of the PI(4,5)P_2_ within the bilayer into domains, reflecting its self-assembly with Syntaxin into nanodomains in vivo (27, 28). The trimeric clusters were disrupted by interface mutations that prevent upright trimer assembly in bilayer-Munc13 cocrystals, suggesting they are identical. Clusters of 3 or more such trimers on PI(4,5)P_2_ domains efficiently bound small vesicles, and such clusters of upright trimers were directly identified on functional giant unilamellar vesicle surfaces reconstituted with Munc13 by cryo-electron tomography.

## Results

### Munc13 Binds Preferentially to the PI(4,5)P_2_-Rich Domains.

To examine the oligomeric state of Munc13-1 on PI(4,5)P_2_-containing bilayers in the absence of DAG we utilized single molecule and statistical methods previously employed to characterize lateral hexamers self-assembling on DAG-containing bilayers (22, 26).

PI(4,5)P_2_ exists in a concentrated form within Syntaxin-containing nanodomains in plasma membranes (28). These domains are therefore the likely targets to which Munc13 binds at the presynaptic plasma membrane. Analogous domains have been shown to self-assemble in a reduced system when the nine amino acid juxta-membrane linker region of Syntaxin-1A is added to PI(4,5)P_2_-containing lipid bilayers (28–30). This positively charged peptide interacts multivalently with negatively charged PI(4,5)P_2_ to assemble the domains. Therefore, we produced similar PI(4,5)P_2_-Syntaxin domains for our Munc13 binding experiments in which a fluorescently tagged Syntaxin peptide was covalently attached to phospholipid, thereby making a Syntaxin lipopeptide, before being incorporated into Small Unilamellar Vesicles (SUVs) which were then used to produce the supported bilayer (*Materials and Methods*, *SI Appendix*, Fig. S1). The SUVs also contained a fluorescent analogue of PI(4,5)P_2_, with a color complementary to that of the Syntaxin peptide to allow simultaneous TIRF imaging.

**Fig. 1.**
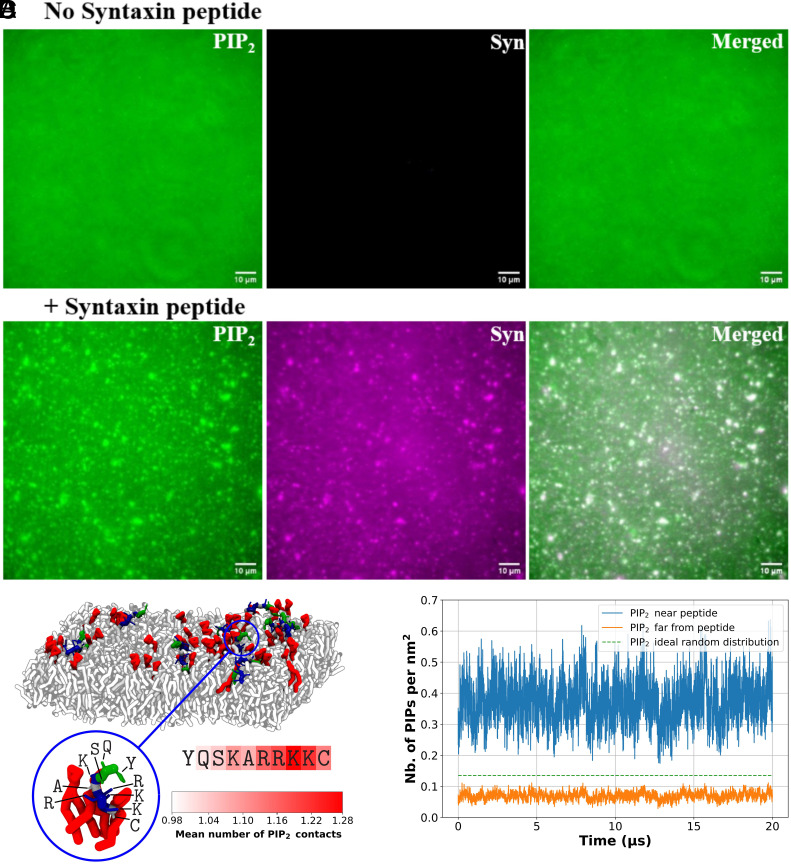
PI(4,5)P_2_ forms domains in lipid bilayer in the presence of Syntaxin lipopeptide. (*A* and *B*) Representative TIRF images of bilayers in the absence (*A*) or presence (*B*) of Syntaxin lipopeptide. The *Left* panels display fluorescent PI(4,5)P_2_, the *Middle* panels show the Syntaxin lipopeptide labeled with CyLyte Fluor5, and the *Right* panels present merged images. (*C*) Aggregation of PI(4,5)P_2_ molecules around a coarse-grained model of the Syntaxin peptide covalently linked to Maleimide-DOPE, obtained from Martini 3 simulations. Representative snapshot from the 20-µs simulation. The zoomed-in view highlights the peptide region interacting with three PI(4,5)P_2_ molecules, together with the identities of the individual residues; the attached Maleimide-DOPE anchor is not shown for visual clarity. The heatmap below the sequence indicates the mean number of PI(4,5)P_2_ contacts per residue throughout the trajectory. The lipid bilayer is composed of DOPC (white), DOPS (light gray), DOPE (dark gray), and PI(4,5)P_2_ (red). The peptide region is colored according to residue type: blue for positively charged, green for polar, and white for nonpolar residues. (*D*) Surface density of PI(4,5)P_2_ molecules over time in membrane regions with PI(4,5)P_2_–lipopeptide clusters (PI(4,5)P_2_ less than 1 nm from a peptide, blue line) and regions without clusters (PI(4,5)P_2_ more than 1 nm from a peptide, orange line). Average surface densities were 0.37 and 0.07 PI(4,5)P_2_ molecules/nm^2^, respectively. For comparison, if the distribution of PI(4,5)P_2_ across the membrane is completely random, the expected density is 0.14 PI(4,5)P_2_ molecules/nm^2^ (green dashed line).

As anticipated (Fig. 1 *A* and *B*), PI(4,5)P_2_ formed optically visible clusters in the presence of the Syntaxin lipopeptide. Without Syntaxin lipopeptide no PI(4,5)P_2_ was observed by TIRF microscopy. The average surface area of coclusters was 1.7 ± 0.2 µm^2^. PI(4,5)P_2_ fluorescence intensities indicate that the fluorescent dyes are slightly quenched in the domains. Estimates of this quenching effect suggest that PI(4,5)P_2_ in coclusters was enriched ~threefold over the bulk bilayer concentration (*Materials and Methods*). Coarse-grained molecular dynamics simulations find a similar enrichment (Fig. 1 *C* and *D* and *SI Appendix*, Fig. S2), supporting the experimental estimate.

Munc13 binds PI(4,5)P_2_ via its C_2_B domain (25). As in previous studies (19, 22, 25), we used the core portion of Munc13-1, comprising the C_1_-C_2_B-MUN-C_2_C domains (residues 529 to 1,735; Munc13C) with a Halo tag fused to its C-terminus (Munc13C-Halo). This truncated form of Munc13-1 is known to be functional in vivo (18, 31). To visualize Munc13C-Halo, we labeled it with an Alexa Fluor 660 Halo ligand whose color was chosen to allow simultaneous imaging with fluorescent PI(4,5)P_2_ and/or tagged Syntaxin lipopeptide in various experiments.

**Fig. 2.**
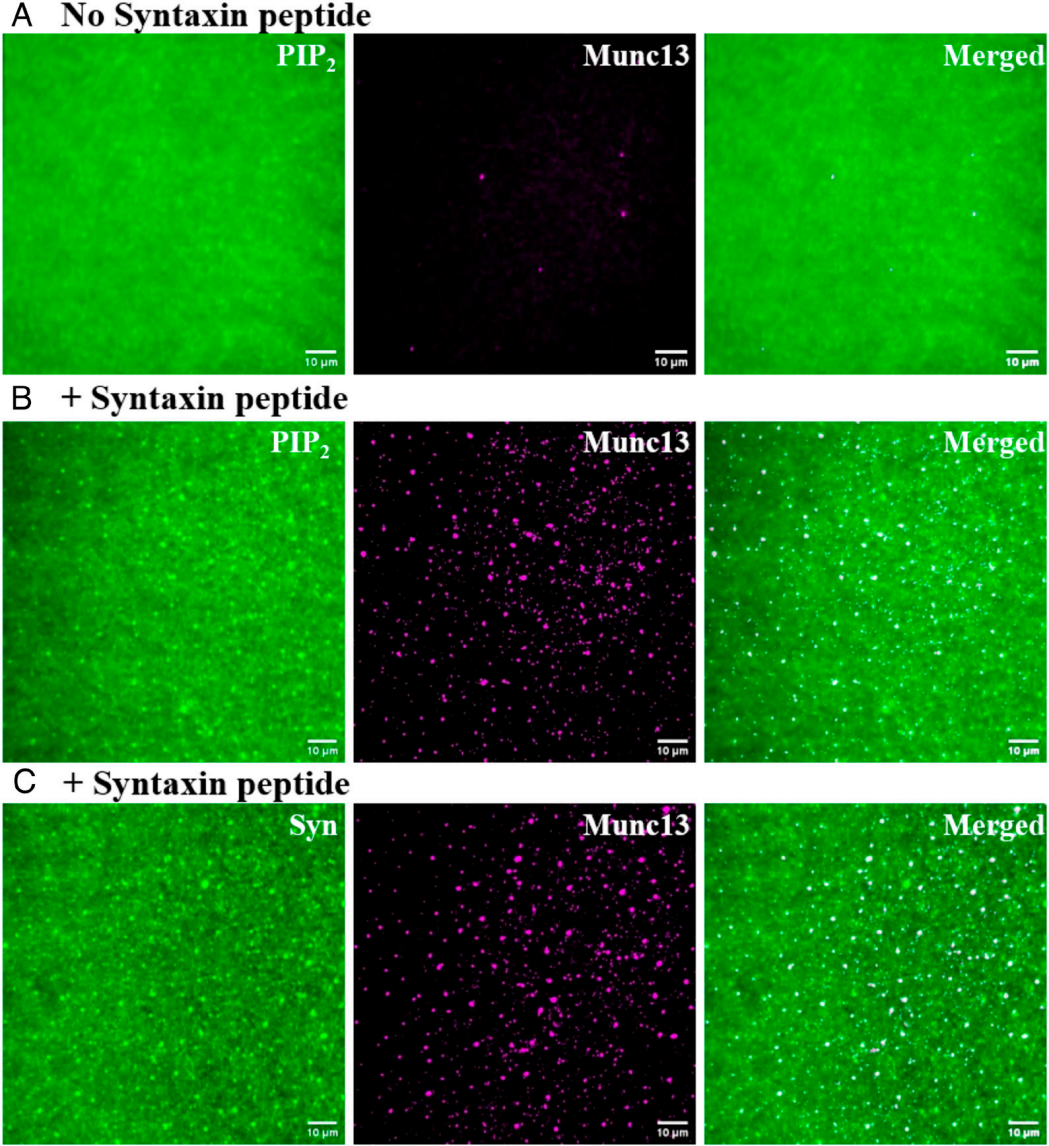
Munc13 is recruited to PI(4,5)P_2_ domains in lipid bilayer in the presence of Syntaxin lipopeptide and forms clusters. (*A* and *B*) Representative TIRF images of PI(4,5)P_2_ and Munc13 bound to bilayers in bilayers without (*A*) or with (*B*) Syntaxin lipopeptide. The *Left* panels display fluorescent PI(4,5)P_2_, the *Middle* panels show Munc13 labeled with Alexa 660, and *Right* panels present merged images. (*C*) Representative TIRF images of Syntaxin lipopeptide and Munc13 bound to bilayers. The *Left* panels display Fluorescein-labeled Syntaxin lipopeptide, the *Middle* panels show Munc13 labeled with Alexa 660, and the *Right* panels present the merged image.

Munc13 Alexa 660, added at 10 nM, bound to the bilayers in clusters which colocalized efficiently with simultaneously imaged domains of fluorescent PI(4,5)P_2_ (Fig. 2*B*). Importantly, Munc13 was not concentrated to bilayers containing the same concentration of PI(4,5)P_2_ but lacking the Syntaxin peptide (Fig. 2*A*). As expected, PI(4,5)P_2_ did not form clustered domains in these bilayers (Fig. 2*A*).

To confirm this result, we repeated this experiment using nonfluorescent PI(4,5)P_2_ and fluorescent Syntaxin juxta-membrane peptide. We observed that Munc13 bound to the resulting bilayer and colocalizes with Syntaxin-1A peptide clusters (Fig. 2*C*). Given the colocalization of Syntaxin peptide clusters and PI(4,5)P_2_ domains (Fig. 1*B*), we propose that the juxtamembrane peptide induces the formation of PI(4,5)P_2_ domains which, in turn, triggers the oligomerization of Munc13.

### Munc13 Predominantly Assembles into Trimers on PI(4,5)P_2_–Rich Domains.

The previous experiments reveal that Munc13 binds efficiently to the PI(4,5)P_2_ within Syntaxin- PI(4,5)P_2_ domains, but poorly to uniformly distributed PI(4,5)P_2_, suggesting that Munc13 binds multivalently. Since each copy of Munc13 has only one PI(4,5)P_2_-binding (C_2_B) domain, the simplest possibility is that Munc13 itself forms oligomeric complexes when bound to the PI(4,5)P_2_-rich domains.

If this is correct, there should be multiple copies of Munc13 in each cluster (representing a nanodomain) and Munc13 should be statistically distributed among the clusters in a manner that reflects the degree of oligomerization. For example, nonoligomerizing monomers would distribute one at a time according to the standard Poisson distribution, whereas trimers would distribute three at a time, etc. In more complex cases, with for example a mixture of monomers and trimers, then the result would be a superposition of the two distributions.

Therefore, we used single molecule counting methods to determine the number of copies of Munc13 in each cluster and the frequency distribution of these copy numbers. The efficiency of Alexa dye labeling of the Halo tagged Munc13 was routinely more than 90% (*Materials and Methods*). As previously described (22, 26), we used systematic low-intensity laser photobleaching to quantify copy numbers. When the protein copy number was less than 5-6, the bleaching profile displayed distinct stepwise decreases, allowing us to determine the exact number of proteins by counting the number and intensity of bleaching steps. For larger copy numbers, the bleaching profile appeared smooth and was best approximated by an exponential decay function. In this case, the copy number *N* can be obtained through, *N* = *I_0_/a*, where *I_0_* is the initial fluorescence intensity before bleaching, and *a* is the unit intensity of a single fluorophore or the average intensity of an average single bleaching step independently determined in the same experiment from the small clusters. *SI Appendix*, Fig. S3 provides examples of bleaching traces of Munc13 clusters.

**Fig. 3.**
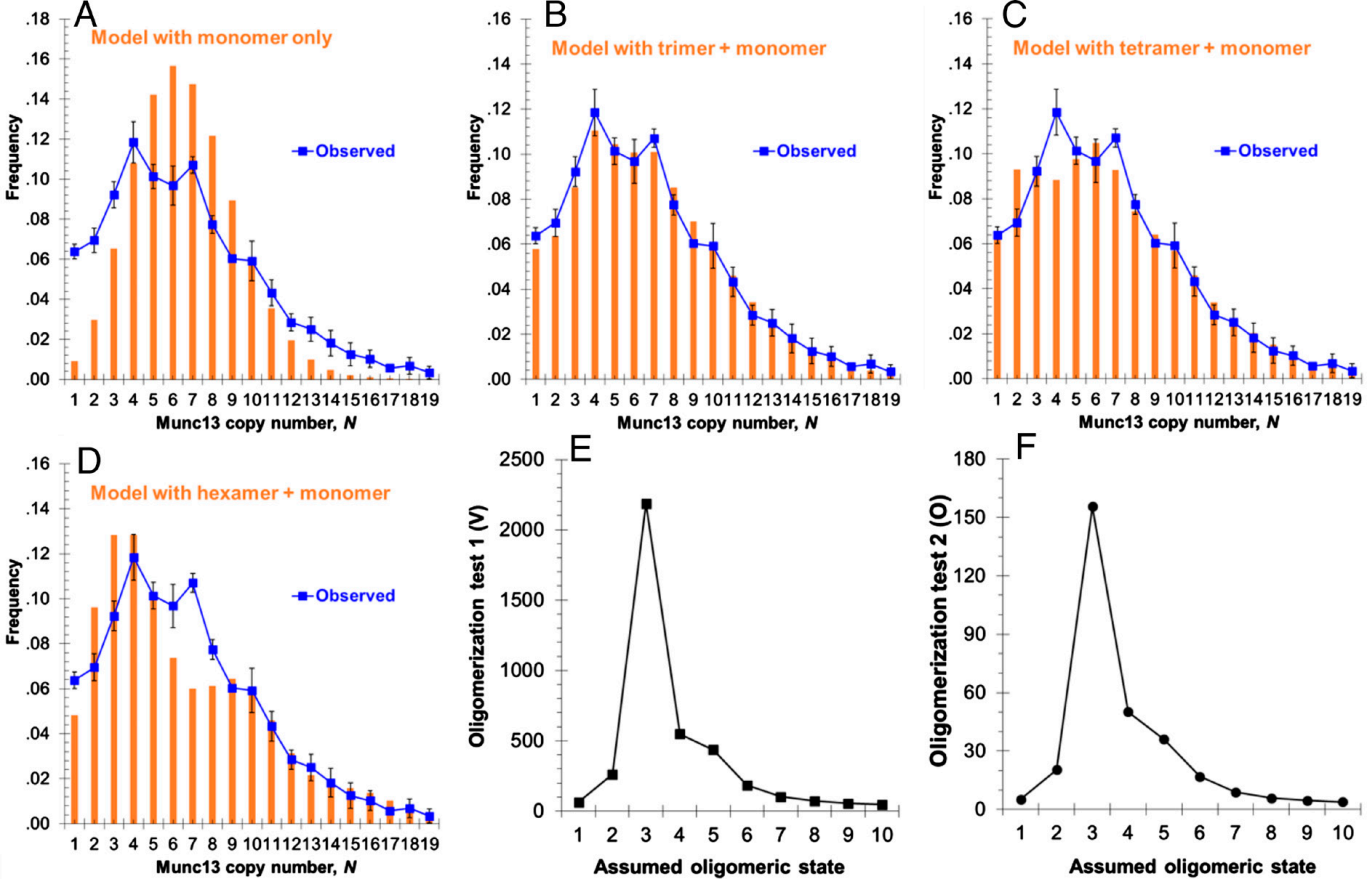
Poisson distribution modeling of oligomers in wildtype Munc13 clusters recruited to PI(4,5)P_2_ domains. (*A*) Single Poisson distribution, assuming all wildtype Munc13 in monomer state; (*B*-*D*) Dual Poisson distribution (monomer + uniform size oligomer), 2-step process: monomer with trimer (*B*), monomer with tetramer (*C*), and monomer with hexamer (*D*). The orange bars represent predicted distribution of the copy number of Munc13 molecules in the clusters, and the blue line is the observed experimental data (sample size *n* = 878). (*E*) Quantification of the inverse of the sum of the square of the difference between measurement and predictions, *V*, described in Eq. **1**, which shows the inverse of the least square of the absolute variation between the measured and predicted size distribution for each assumed oligomeric state of wildtype Munc13 (*Materials and Methods*). (*F*) The *O* value of the Poisson modeling, described in Eq. **2**, as function of the assumed oligomeric state of wildtype Munc13. Larger *V* and *O* values correspond to better fits of the prediction with observed data.

The resulting histogram of Munc13 copy numbers per cluster (Fig. 3*A*) revealed a broad distribution, ranging from 1 to 19 molecules. Notably, peaks were observed at 4, and seven copies of Munc13. In contrast, our previous study of Munc13 clusters on DAG (and PI(4,5)P_2_)-containing bilayers showed peaks at 8 and 14 copies (22). Hence, the organization of Munc13 in PI(4,5)P_2_ domains differs significantly from its arrangement in the presence of DAG.

As before with DAG, further insight required statistical analysis (22). The observed copy number in a given cluster, *N*, represents the sum of Munc13 monomers and oligomers within the cluster. For example, an optically discrete cluster containing four copies of Munc13 (*N* = 4) could consist of four noninteracting monomers freely diffusing over a common lipid domain. Alternatively, it could comprise a combination of one monomer and one trimer, or two dimers or even a single tetramer.

Assuming a random distribution of monomers and oligomers of fixed size, we previously found that Munc13 forms hexamers in the presence of DAG-containing domains (22). We applied a similar approach to analyze the copy number histogram in Fig. 3. First, we tested the null hypothesis, which assumes that Munc13 molecules in a cluster exist only as independent, noninteracting monomers, with an average copy number of molecules per cluster. Under this assumption, the frequency distribution of Munc13 monomer copy number, *m*, should follow the standard Poisson distribution. Experimentally, we measured an average of 6.6 Munc13 per cluster. The observed distribution deviated significantly from that predicted by the standard Poisson distribution with an average 6.6 (Fig. 3*A*). This discrepancy indicates that a simple model of randomly distributed monomers does not accurately describe Munc13 clustering, i.e., qualitatively we can see that Munc13 is oligomerizing to one or another degree within the PI(4,5)P_2_-rich domains.

Next, we tested whether the experimental data could be better explained by a mixture of randomly distributed monomers and fixed-size *K*-mers (dimers in one model; trimers in a second model; etc.) within each cluster. In this case, the *K*-mers and the monomers each followed their own independent Poisson distribution.

The probability distribution for a cluster of size *N* can then be predicted as previously (22) and experimental data provide the average number of monomers and *K*-mers per cluster, $<m>$ and $<k>$ respectively. This allowed us to precisely calculate the distributions of copy numbers, *N*, for each *K*-mer. $<m>$ is obtained by fitting the histogram for Munc13 copy number below *K*, since, by definition, there cannot be any *K*-mer in such clusters. Then, $<k>$ is calculated without any fit from the mean number of Munc13 copies per cluster (22). The final predicted distribution is obtained from the sum of the distribution of monomers and *K*-mers.

Using this approach, we predicted the copy number distribution (frequency versus *N*) and compared these predictions with experimental data (Fig. 3 *B*–*D* and *SI Appendix*, Fig. S4) for *K* = 1 to 10. The experimental results (dashed line in Fig. 3*B*) closely fit the model only for *K* =3 (trimers), with $<m>=2.2$ and $<k>=1.5$. Specifically, the two peaks (*N* = 4 and *N* = 7) closely matched the features predicted by the trimeric model. In contrast, models with *K* = 2, *K* = 4, or *K* = 6, did not align with these observed peaks (Fig. 3 *C* and *D* and *SI Appendix*, Fig. S4).

To confirm the most likely degree of oligomerization, we used two parameters, *V* and *O* (*Materials and Methods*), which provide an unbiased measure of fit between the observed and predicted cluster size distributions. For each *K-mer*, these parameters quantitatively compare the predicted and observed probabilities. Higher *V* or *O* values indicate a better fit. As shown in Fig. 3 *E* and *F*, both parameters reached their maximum at *K* = 3.

Altogether, from these single molecule counting data and statistical analyses we conclude that Munc13 predominantly self-assembles into trimers when bound to PI(4,5)P_2_-enriched domains.

### Targeted Mutations That Destabilize the Upright Trimers Observed in Crystals Also Disrupt the Self-Assembled Putative Trimers.

To independently test this conclusion, and further to determine if these are the same as the upright trimers present in the Munc13-bilayer cocrystal, we employed mutations that target both sides of the unique intersubunit interface that stabilizes the trimer. These upright trimer interface mutations disrupt the upright trimers in the cocrystal without affecting Munc13 lateral hexamer assembly while also preventing synaptic vesicle priming in the functional reconstitution of ready-release vesicles and also preventing synaptic transmission in vivo in *C*. *elegans* (15). If the trimers inferred from copy number distributions are upright trimers, they too should be disrupted, and the copy number distributions altered, by trimer interface mutations.

**Fig. 4.**
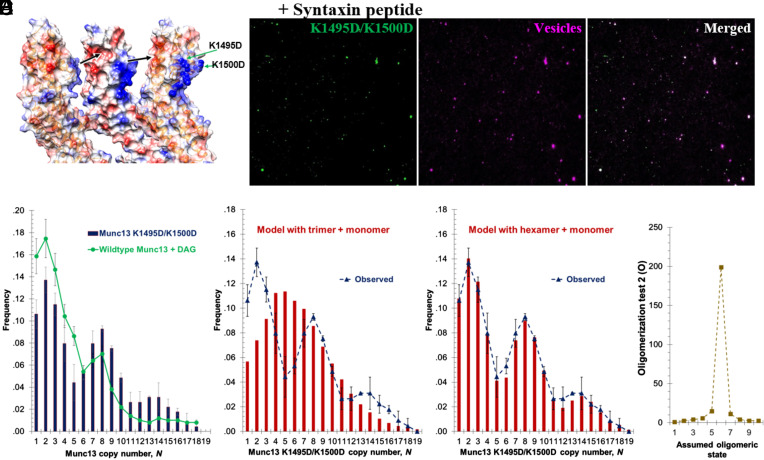
Mutations in upright trimer interface affect protein organization, and cluster size distribution. (*A*) Close-up view of the upright trimer interface of Munc13 oligomer in ribbon representation (adapted from ref. 25). The interface contains two regions: positively charged (blue colored patch) and negatively charged (red colored patch). The black arrows indicate the electrostatic interactions between these two patches. The green arrows refer to the mutated residues (K1495D, K1500D) in our experiments. (*B*) Representative TIRF image of Munc13 trimeric interface mutant labeled with Alexa 488 on lipid bilayer membrane with the Syntaxin lipopeptide; and of vesicles captured by the same Munc13 interface mutant (vesicles labeled with Atto647N). The *Left* panel displays Munc13, the *Middle* panel shows bound vesicles, and the *Right* panel presents the merged image. (*C*) Distribution of the copy number of Munc13 trimeric interface mutant, K1495D/K1500D, in the clusters (dark blue bars) (sample size *n = 226*). The green line is the size distribution of the wildtype Munc13 recruited to DAG domains, which serves as a reference (22). (*D*) The predicted size distribution of the clusters of the mutant, K1495D/K1500D, using dual Poisson distribution (monomer + uniform size oligomer), assuming the oligomeric state corresponds to trimers (dark red bars), which does not fit the experimental data (dark blue dashed line). (*E*) The predicted size distribution of the clusters (dark red bars) using dual Poisson distribution, assuming the oligomeric state corresponds to hexamers, fits well the experimental data (dark blue dashed line). (*F*) The *O* value of the Poisson modeling, described in Eq. **2**, as function of the assumed oligomeric state of the Munc13 trimeric interface mutant.

The trimer interface (Fig. 4*A*) involves electrostatic interactions between a positively charged surface on each MUN domain (residues K1494, K1495, and K1500) with a negatively charged surface on the neighboring MUN domain (residues D1358 and D1369). Notably, this charged interface is well conserved across vertebrates (15). We tested a charge-reversing double mutation (K1495D/K1500D) which has no effect on the intrinsic chaperone activity of soluble monomers of Munc13, whereas cryoelectron tomography of this mutant revealed that the lattice organization of the upright trimers was substantially abolished (15).

Similar to the wild-type Munc13, this trimeric interface Munc13 mutant, Munc13^K1495D/K1500D^, assembles into clusters on the bilayer membrane in the presence of PI(4,5)P_2_ and Syntaxin juxtamembrane peptide with comparable overall membrane binding (Fig. 4*B*). However, the distribution of protein copy numbers among the clusters was markedly different (Fig. 4*C*). The characteristic peaks of wild-type Munc13 peaks at N = 4 and N = 7 were absent, and were replaced by new peaks at N = 8 and N = 14. This result independently confirms our conclusion from the statistical analysis that the PI(4,5)P_2_-rich domains predominantly contain trimers of Munc13, and strongly suggests that they assemble via the same intersubunit interface that stabilizes the known upright trimer.

Peaks at N = 8 and 14 were previously observed for lateral hexamers that assembled on DAG-rich domains along with remaining monomers (22), suggesting that this trimer interface mutant assembles primarily into lateral hexamers. In fact, the distributions of Munc13^K1495D/K1500D^ [on PI(4,5)P_2_-rich domains in the absence of DAG] and Munc13^wt^ lateral hexamers [on DAG-rich domains that also contain PI(4,5)P_2_ (21)] are similar (Fig. 4*C*). Further statistical analysis confirmed that the best fit for Munc13^K1495D/K1500D^ is with *K* = 6 (Fig. 4 *D*–*F* and *SI Appendix*, Fig. S5). That lateral hexamers should predominate, even in the absence of DAG, when the alternative upright trimer is destabilized, is not surprising because this is what was observed in cocrystals (15) and it suggests that they are in an equilibrium.

### Upright Trimers Are Directly Observed by Cryo-Electron Tomography of Functionally Reconstituted Membranes.

**Fig. 5.**
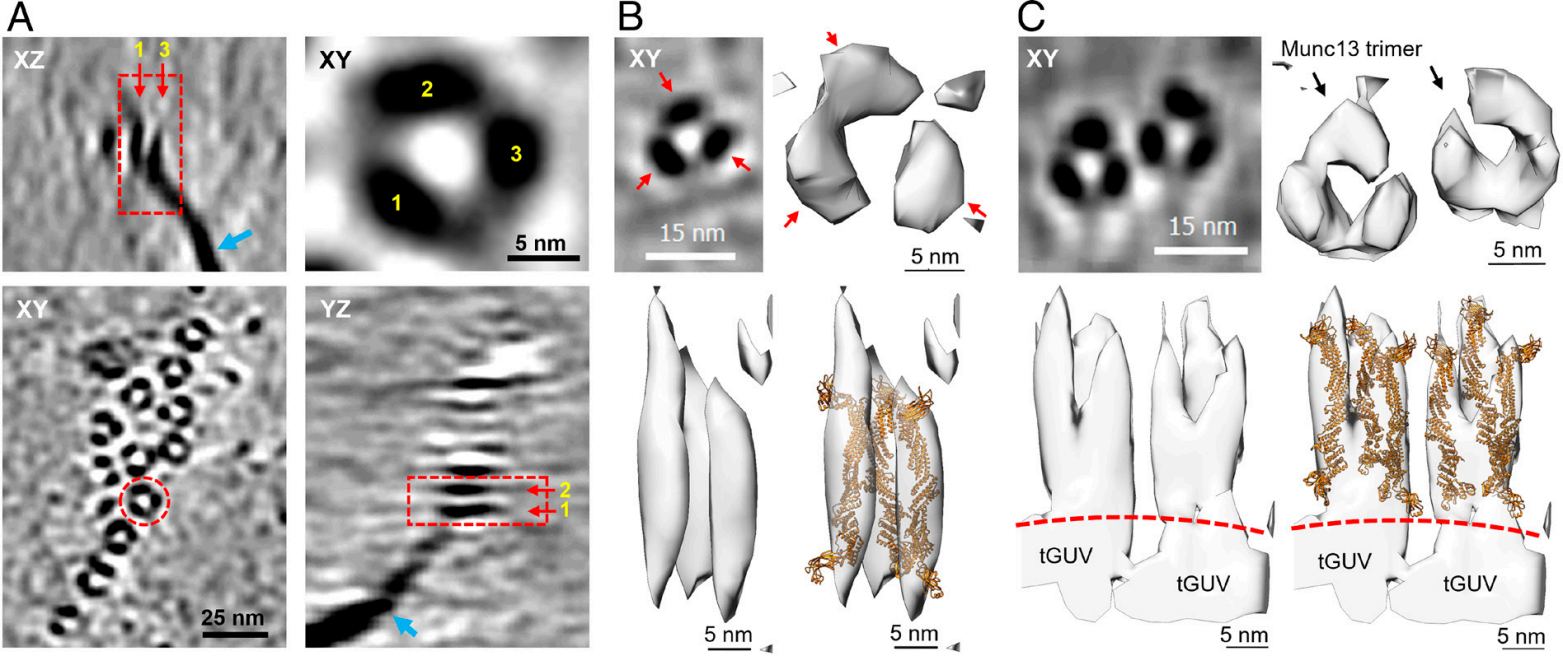
Assemblies of upright Munc13 trimers on negatively charged GUVs visualized by cryo-ET. (*A*) Tomographic XY slice showing a cluster of triangular Munc13C assemblies on the GUV surface. *Inset* (*Top Right*) magnifies a selected triangular structure (dashed red circle), with vortices numbered in yellow. XZ and YZ slices (*Top Left* and *Bottom Right*) depict protein densities protruding from the GUV membrane (red boxes), while blue arrows indicate the flattened GUV edges. (*B*) Example of an isolated Munc13C trimer. Single XY tomogram slice (*Top Left*) and 3D surface renderings in top-view (*Top Right*) and side-view (*Bottom Left*) are shown. The atomic model of the C_1_-C_2_B-MUN-C_2_C fragment [PDB 7T7X, orange, (1)] is docked into the volume (*Bottom Right*). (*C*) Example of a cluster of two Munc13C trimers. Tomogram slice (*Top Left*) and 3D surface renderings in top-view (*Top Right*) and side-view (*Bottom Left*) are shown. The atomic model [PDB 7T7X, orange, (25)] is fitted into the volume (*Bottom Right*). The dotted red line outlines the GUV membrane. Docking was performed with the C_2_B domains oriented toward the membrane and the C_2_C domains facing outward, poised for vesicle capture. Figues were generated from Bin8 tomograms at 2.6 nm pixel size.

If the foregoing is correct, it should be possible to observe upright trimers assembling on the plasma membrane equivalent used in the physio-mimetic cell-free reconstitution of vesicle priming for ready release (19). To test this, we incubated wildtype Munc13 with the reconstituted Giant Unilamellar Vesicles (GUVs) routinely used to form the suspended bilayers mimicking presynaptic plasma membrane and examined the products by cryoelectron tomography (Fig. 5). These GUVs contain both full-length Syntaxin-1 (as the 1:1 Syntaxin–Munc18 complex), palmitoylated SNAP25 (19) and 6 mol% PI(4,5)P_2_ which are known to assemble into PI(4,5)P_2_ -rich domains (28). The reconstructed tomograms showed rod shaped densities corresponding to individual Munc13 tethers sticking out of the tGUV surface (*SI Appendix*, Fig. S6). These Munc13 tethers appear to be highly flexible as monomeric units consistent with similar observations from other research groups in the past (17, 32).

In addition, we frequently observed triangular protein structures on the surface of the GUVs (Fig. 5*A* and Movie S1). These triangular assemblies appeared both in clusters and as isolated units (*SI Appendix*, Fig. S7). Detailed analysis of orthogonal slices through the triangular structures revealed that each vertex comprises a long, tether-like protein extending up to ~26 nm from the GUV surface (Fig. 5*A*). Given that wildtype Munc13 is the only protein in the mixture with these dimensions, it is highly likely that each trimer consists of three Munc13 molecules. Hence, we manually docked the atomic model of the upright conformation of Munc13-1 C_1_-C_2_B-MUN-C_2_C in [PDB 7T7X, as described by Grushin et al. (25)] into 3D surface renderings of the subtomograms of isolated and clustered triangular assemblies (Fig. 5 *B* and *C*). Our structural analysis suggests that three upright Munc13 molecules constitute these triangular assemblies (Movies S2 and S3). Hence, wildtype Munc13 forms upright trimers that are not sandwiched between two lipid bilayers.

The upright trimers in the Munc13-bilayer cocrystal interact in pairs via antiparallel contacts between their membrane-proximal (DAG-binding) C_1_ domains (25). The atomic model of Munc13C trimer [PDB 7T7R, described by Grushin et al. (25)] did not dock well into the 3D surface renderings of the triangular assemblies suggesting that the clusters of upright trimers assembling on the GUVs are bonded differently. However, based on the docking of the atomic model of Munc13 on these densities, the Munc13 trimers are attached to the GUV bilayers by their C_2_B domains while the synaptic vesicle-binding C_2_C domains (17, 26) were positioned~20 nm above the GUV surface, suggesting a conformation that would facilitate vesicle capture, whether by individual upright trimers or by clusters of them.

Munc13 monomers are flexible and are observed in several conformations whereas the trimers are locked in a more rigid upright conformation. This suggests that when the surface concentration of Munc13 is high, the flexible monomers come together and settle into a more ordered trimeric state. However, confirmation of this will require further studies with detailed structural analysis including subtomogram averaging of the trimeric structures.

### Vesicles Bind to Clusters of 3 or More Upright Trimers of Munc13 in the PI(4,5)P_2_-Rich Domains.

**Fig. 6.**
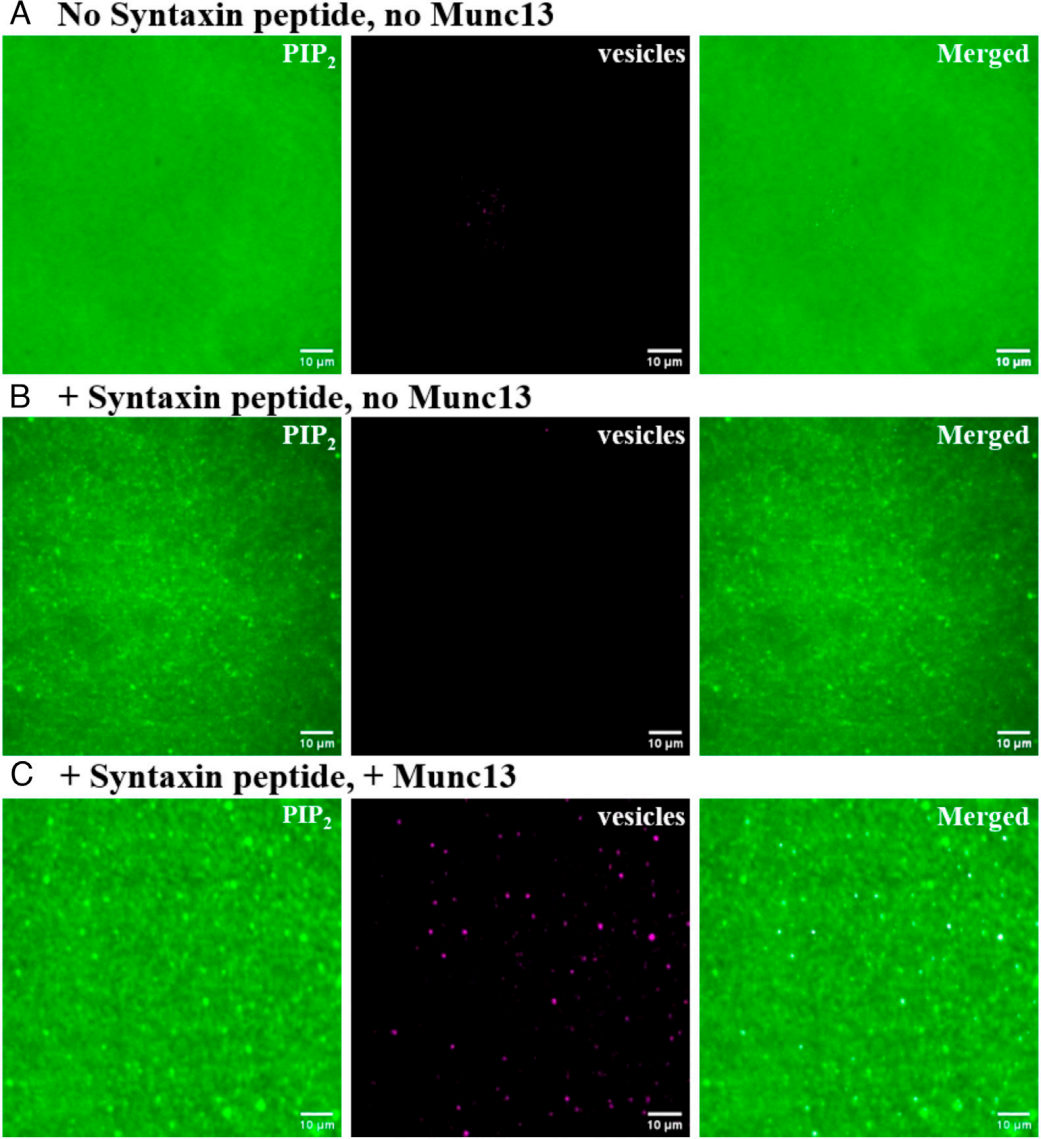
Vesicle recruited to clustered PI(4,5)P_2_ domains in the presence of Syntaxin peptide and Munc13. (*A*-*C*) Representative TIRF images of fluorescent PI(4,5)P_2_ in bilayers and vesicles bound to bilayers without (*A*) or with (*B* and *C*) Syntaxin lipopeptide. After lipid bilayers were formed on the coverslip, they were first incubated with buffer (*A* and *B*) or Munc13 solution (*C*), and then incubated with vesicle solution (labeled with Atto647N). The *Left* panels display fluorescent PI(4,5)P_2_, the *Middle* panels show captured vesicles, and the *Right* panels present the merged images.

To test the ability of the self-assembled upright trimers to capture vesicles, we exposed the supported bilayers to protein-free SUVs (69 mol% DOPC, 30 mol % DOPS and 1 mol% DOPE-Atto647N). Unbound vesicles were washed away with buffer. We then imaged both PI(4,5)P_2_ and SUVs to assess colocalization (Fig. 6).

Indeed, when PI(4,5)P_2_ and the lipid-linked Syntaxin-1 peptide and Munc13 were all present, SUVs were bound. Merged images confirmed that these vesicles colocalized with PI(4,5)P_2_ domains (Fig. 6*C*). Omitting any of Munc13 (Fig. 6 *A* and *B*) or the Syntaxin lipopeptide (Fig. 6*A*) prevented vesicle binding.

**Fig. 7.**
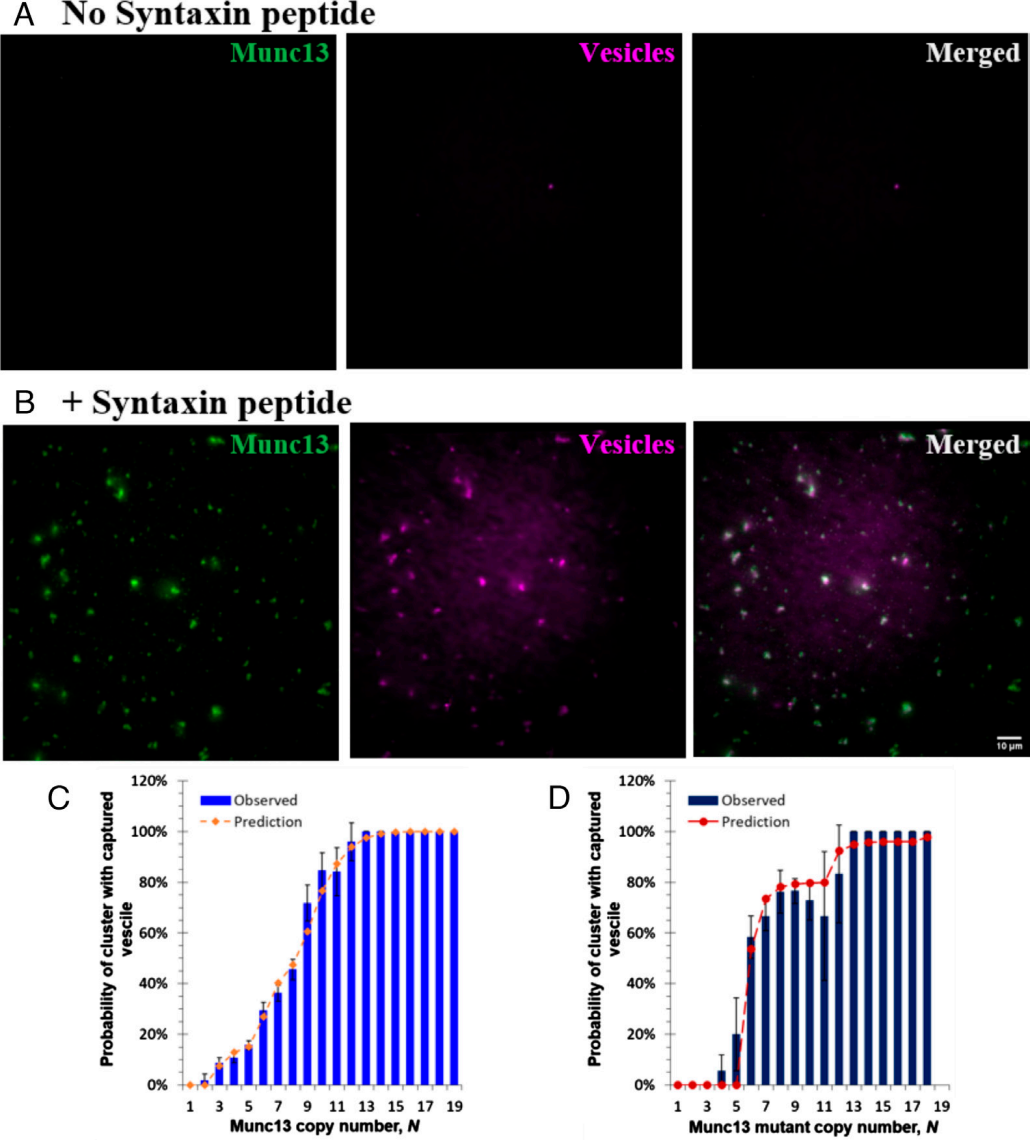
Vesicle binding by Munc13 clusters in PI(4,5)P_2_ domains. (*A* and *B*) Representative TIRF images of wildtype Munc13 labeled with Alexa 488 on lipid bilayers and vesicles bound to bilayers without (*A*) or with (*B*) Syntaxin lipopeptide. The *Left* panels display Munc13, the *Middle* panels show bound vesicles, and the *Right* panels present the merged image. (*C*) Probability of vesicle capture by the clusters of wildtype Munc13 as a function of the copy number of the clusters (sample size *n* = 878). The observed experimental data (blue bars) are consistent with the prediction (dashed orange line) in which we assume the vesicle capture probabilities of 1 trimer, 2 trimers, or 3 or more trimers are 0.17, 0.55, and 1, respectively. (*D*) Probability of vesicle capture by the clusters of Munc13 upright trimeric interface mutant as a function of the mutant copy number in the cluster (sample size *n* = 226). The observed experimental data (dark blue bars) can be predicted (dark red dashed line) by modeling the clusters of the trimeric interface mutant as hexamer and assuming 1 hexamer has a probability of 0.8 to capture a vesicle, and 2 or more hexamers have the probability of 1 for vesicle capture.

To confirm the expected role of the Syntaxin juxtamembrane peptide in more detail, we repeated the vesicle binding experiments now using fluorescently labeled Munc13 and only nonfluorescent PI(4,5)P_2_. Here, we compared the colocalization of Munc13 clusters and SUVs bound to bilayers in the absence (Fig. 7*A*) or presence (Fig. 7*B*) of Syntaxin juxtamembrane peptide. We found that Munc13 clusters and SUVs colocalized only when the Syntaxin peptide was present. These findings confirm the critical role of the Syntaxin peptide in coassembling with PI(4,5)P_2_ into microdomains, which in turn recruit Munc13 trimers that then can capture vesicles from solution.

The PI(4,5)P_2_ -rich domains present the vesicles with an array of different targets for binding, as they can contain only monomers (when *N* ≤ 2) or a mixture of monomers and upright trimers (when *N* ≥ 3). When N = 19 (the largest number of Munc13s occasionally observed in a cluster) there could be a maximum of six upright trimers. Therefore, the number of upright trimers per microdomain will range from none (*N* ≤ 2) to 6. To establish the effect of Munc13 copy number on the efficiency of vesicle capture, we quantified Munc13 and SUV binding simultaneously, and from these data, we calculated (see *Materials and Methods* for details) the probability that a microdomain binds one or more vesicles as a function of the number of copies of Munc13 it contains (Fig. 7*C*).

There is no meaningful vesicle binding when N = 1 or 2 (though it is easy to find domains containing only 1 to 2 Munc13 molecules; see Fig. 3 for example). This suggests that a single upright trimer is the basic unit for vesicle binding. The probability distribution is nearly saturated at *N* ≥ 9, and appears to rise in three steps altogether. The simplest interpretation is that these steps each represent an additional upright trimer, and that vesicle capture is optimal when there are 3 upright trimers.

To test this idea more rigorously, we developed a statistical model to predict the probability to capture a vesicle by a certain cluster with copy number *N* (*Materials and Methods*), using the data from the above Poisson analysis to estimate the chance that each cluster contains 1, 2, 3, or more trimers. The predicted vesicle binding probability as a function of *N* closely matches the measured distribution (Fig. 7*C*) when the probabilities of capturing a vesicle by 1, 2, and 3 or more trimers are 0.17, 0.55, and 1, respectively. This confirms that vesicles are indeed captured by Munc13-1 trimers and that 3 trimers guarantee the capture of a vesicle.

**Fig. 8.**
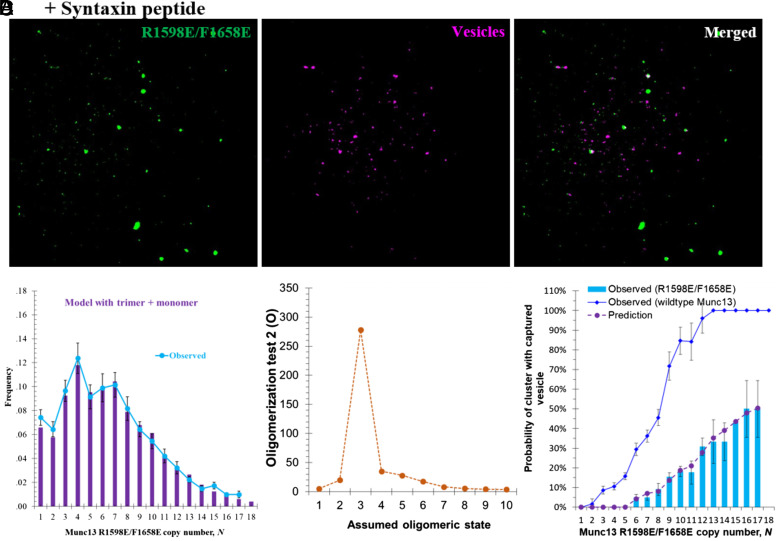
Vesicle binding efficiency of Munc13 trimers is impaired by mutations on the C_2_C domain. (*A*) Representative TIRF image of Munc13 C_2_C R1598E/F1658E mutant labeled with Alexa 488 on lipid bilayer membrane containing 71% DOPC, 25% DOPS, 2% PI(4,5)P_2_, and 1.3% bare Maleimide-DOPE lipid & 0.7% Syntaxin peptide-Maleimide-DOPE; and image of captured vesicles by the same Munc13 C_2_C mutant (vesicles labeled with Atto647N). The *Left* panel displays Munc13 Alexa488, the *Middle* panel shows bound vesicles, and the *Right* panel presents the merged image of the previous two. (*B*) Distribution of the copy number of Munc13 C_2_C R1598E/F1658E mutant molecules in the clusters (cyan solid line) (sample size *n* = 404). The purple bars are the predicted size distribution of the clusters using dual Poisson distribution (monomer + uniform size oligomer), assuming the oligomeric state are trimers, which fits well the experimental data. (*C*) Variation of the *O* value of the statistical modeling, described in Eq. **2**, as a function of the assumed oligomeric state of the Munc13 C_2_C R1598E/F1658E mutant. Larger *O* value indicates a better fit of the observed data by the predictions. (*D*) Probability of vesicle capture by the clusters of Munc13 C_2_C R1598E/F1658E mutant as a function of the C_2_C mutant copy number (sample size *n* = 404). The observed experimental data (cyan bars) can be predicted (purple dashed line with circles) by modeling the clusters of the C_2_C mutant as trimer and assuming the probabilities to capture a vesicle for 1, 2, 3, 4, and 5 trimers are 0, 0.09, 0.23, 0.43, and 0.53, respectively. The blue solid line with diamonds represents the probability of wildtype Munc13 clusters, which serves as a reference.

To be certain that our in vitro vesicle binding assay faithfully reflects the in vivo process, we tested the effect of a double mutation (R1598E/F1658E) of the C_2_C domain which impairs vesicle docking, priming, and Ca^2+^ triggered release by substantially reducing the size of the readily releasable vesicle pool (17). While not affecting Munc13 oligomerization (Fig. 8 *A*–*C* and *SI Appendix*, Fig. S8), these C_2_C domain mutations profoundly reduced vesicle binding (Fig. 8*D*). The probabilities of vesicle capture by 1, 2, and 3, trimers to capture a vesicle were now reduced to 0, 0.09, and 0.23, respectively.

These results suggest a pathway in which three Munc13-1 molecules preassemble into an upright trimer and likely further cluster (Fig. 5) on PI(4,5)P_2_-rich Syntaxin–Munc18-containing microdomains, comprising the basic units for vesicle capture. If this model is correct, disrupting the upright trimer should eliminate the “units of 3” cooperativity observed for vesicle binding (Fig. 7).

To test this, we repeated the vesicle capture experiment with the upright trimer interface mutant Munc13^K1495D/K1500D^ which assembles mainly into lateral hexamers rather than upright trimers (Fig. 4). Indeed, the “units of 3” behavior is abolished, and replaced by a striking “units of 6” behavior (Fig. 7*D*) as observed previously (22) for lateral hexamers produced by DAG from wild-type (rather than from trimer-interface mutant) Munc13. Evidently, vesicles are now captured by the hexamers in the mutant clusters.

## Discussion

The upright trimer of Munc13 was identified as a building block (along with lateral hexamers) in a protein cocrystal that fortuitously assembles between the surfaces of large unilamellar acidic phospholipid bilayer vesicles (25). Genetic studies, in which the interface stabilizing the trimer is mutated, imply that the trimer participates critically in synaptic vesicle priming for ready release, whether in vivo in worms or in vitro in a functional reconstitution (15).

In the present study we demonstrate that these upright trimers can exist outside the crystal as discrete self-assembling entities capable, especially in cooperation with each other, of capturing vesicles. Specifically, Munc13 distributes among PI(4,5)P_2_ -rich microdomains in units of 3, suggesting it forms trimers; interface mutations that disrupt the upright trimers but not lateral hexamers in cocrystals (15) also cause Munc13 to distribute among microdomains as hexamers instead of trimers; trimeric densities are directly observed by cryo-electron tomography and are well fitted by three molecules of the known upright Munc13 conformation from the cocrystal (25); Munc13 trimers self-assembling on microdomains capture artificial phospholipid vesicles in cooperative units of 3 copies each; and finally, the trimers use the same amino acid residues (in the C_2_C domain) that carry out this function in vivo (17).

These results provide additional strong evidence in support our model (10, 15, 25) for vesicle priming in which synaptic vesicles, initially captured by clusters of Munc13 upright trimers, transition to lateral hexamers, bringing the vesicle close enough to the plasma membrane for the first six SNAREpins to assemble within the hexamer. This transition is favored in vitro by DAG (22), which is a product of PI(4,5)P_2_ hydrolysis by neuronal phospholipase C triggered by with Ca^2+^ influx (33–35), signaling the need to replace consumed synaptic vesicles with newly primed ones. We propose that the PI(4,5)P_2_-to-DAG cycle at the synapse contributes, likely along with other factors, to driving the conformational changes in Munc13, playing a critical role in priming synaptic vesicles.

Since the Munc13 trimer and hexamer identified with single molecular analysis in this and prior work (22), respectively, appear identical to the upright trimer and lateral hexamer observed by Cryo-electron tomography (25), the vesicle would be positioned approximately 20 nm away from the membrane when bound to the trimer and 12 nm away when bound to the hexamer. These transitions successively bring the vesicle closer to the plasma membrane, likely enabling the two successive waves of SNAREpin assembly that are observed when priming is reconstituted (15, 23).

Another key finding is that the formation of the upright trimer requires that PI(4,5)P_2_ not only be present but further must also be clustered into domains to enable Munc13 to bind. In our experiments the microdomains are created by including the cationic juxtamembrane peptide of Syntaxin-1A attached to a phospholipid. As previously shown (27, 28), this peptide effectively substitutes for the entire Syntaxin molecule in clustering the PI(4,5)P_2_ into domains that mimic the domains in which Syntaxin is clustered in native plasma membranes (28). This requirement for PI(4,5)P_2_-rich Syntaxin-1A domains effectively targets docking synaptic vesicles ensures the presence of abundant quantities of this t-SNARE even when it is dilute in the plasma membrane overall.

As a final point, it may seem surprising that Munc13 trimers were not detected in previous studies (17, 32, 36, 37). However, we now understand that the formation of stable Munc13 trimers requires specific conditions: highly negatively charged membranes, the presence of the C_2_C domain of Munc13, and the absence of Synaptotagmin in the vesicle. The importance of Synaptotagmin exclusion is not detailed in the present manuscript, which focuses exclusively on the trimeric state, but will be addressed in a forthcoming study. Since these requirements were previously unknown, previous studies did not fulfill them. As noted in the Introduction, upright trimers are expected to be transient and possibly short-lived; therefore, it is unsurprising that they were not previously detected under conditions in which the necessary requirements were not met. Nonetheless, the vesicle-priming pathway appears to require upright trimers, as mutations at the upright trimer interface impair vesicle priming in vitro and synaptic transmission in worms (15).

## Materials and Methods

### Protein Constructs, Expression, and Purification.

Similar to our previous report (22, 26), the expression plasmids: His_12__PreScission -C_1__C_2_B_MUN_C_2_C_tev_Halo, the upright trimer interface mutant His_12__PreScission -C_1__C_2_B_MUN_C_2_C_tev_Halo (K1495D K1500D), and the C_2_C mutant His_12__PreScission -C_1__C_2_B_MUN_C_2_C_tev_Halo (R1598E F1658E), were produced by cloning rat Munc13-1 residues 529 to 1,735, respectively, to a pCMV-AN6 plasmid. Munc13-1 residues 1,408-1,452 were deleted and residues EF were added in their place (38). A short linker sequence containing a TEV cut site was subcloned in after residue 1735, followed by the Halo tag. The QuickChange mutagenesis kit (Agilent Technologies) was used to generate the mutations.

The resulting plasmids were amplified with maxi prep using QIAGEN Plasmid Maxi kit and were used to transfect Expi293F™ human cells. Proteins were expressed with Expi293™ expression system following the manufacturer’s protocol, and then purified as described before (22, 26, 39, 40).

### Protein Labeling.

The Halo tagged Munc13 proteins were labeled by incubating the proteins with Alexa488 or Alexa660 conjugated with Halo ligand from Promega, as described before (9, 14, 22, 26). Unreacted dye was removed by using the illustra NAP-5 and NAP-10 (Cytiva GE Healthcare) or fluorescent dye removal columns (Thermo Scientific) (14, 22, 26).

The labeling efficiencies of WT Munc13-Halo and the mutants are more than 90%, similarly to previous report (22, 26).

### Chemicals.

As previously described (22, 26), chemicals for buffer and protein purification were purchased from Sigma-Aldrich and Thermo Fisher Scientific. Lipids including TopFluor lipids were purchased from Avanti Polar Lipids. 2-Dioleoyl-sn-gylcero-3-phosphoethanolamine ATTO 647N (DOPE-Atto647N) was from ATTO-Tec. The plasmid maxi prep kit was from QIAGEN. Chemicals and reagents for the Expi293 expression system were purchased from Thermo Fisher Scientific. Fluorescent dyes for labeling proteins were purchased from Thermo Fisher Scientific and Promega Corporation.

All aqueous solutions were prepared using 18.2 MΩ ultrapure water.

### Syntaxin Lipopeptide Synthesis.

Syntaxin lipopeptide was prepared immediately before each experiment. DOPE-maleimide lipid was vacuum dried, resuspended in 100 µL buffer containing 50 mM HEPES, 140 mM KCl, 1 mM TCEP, and 20% OG, and Syntaxin-1A juxtamembrane peptides (purchased from AnaSpec: see *SI Appendix*, Fig. S1 for sequence) solution was subsequently added at a 3:1 maleimide to peptide ratio. After 90 min at room temperature, 100 mM mercaptoethanol was added to reach a final concentration of 4 mM and quench unreacted maleimide. This Syntaxin lipopeptide solution was then ready to be incorporated into SUVs via detergent resuspension of lipid films.

### SUV Formation.

Two protocols were used to make SUVs: extrusion and resuspension in presence of detergent followed by dialysis. SUVs used for lipopeptide-free bilayers were indifferently prepared with any of the two protocols that led to similar results. SUVs with lipopeptides were prepared by detergent resuspension. SUVs that were captured by Munc13 clusters were prepared by extrusion.

#### Extrusion protocol.

We first used an Avestin miniextruder (22, 26) to produce lipopeptide-free liposomes. To make extruded SUVs for preparing bilayers, DOPC, DOPS, PI(4,5)P_2_, TopFluor™ PI(4,5)P_2_ or TopFluor™ TMR PI(4,5)P_2_, and DOPE-maleimide were mixed at a 71:25:1.5:0.5:2 mol ratio to reach 1.5 μmole total lipid, and dried by Nitrogen flow and then vacuum drying. The lipid films were then resuspended, treated with freezing and thawing cycles, and finally extruded, similarly to previous reports (22, 26).

To make the SUVs for vesicle capture experiment, 69 mol% DOPC, 30 mol% DOPS, and 1 mol% DOPE-Atto647N were mixed to reach a 1.5 μmole total lipid. SUVs were produced by extrusion similarly as above.

#### Detergent resuspension protocol.

1.5 µmol DOPC, DOPS, PI(4,5)P_2_, TopFluor™ PI(4,5)P_2_ or TopFluor™ TMR PI(4,5)P_2_ (71:25:1.5:0.5 in mol %) was dried under a nitrogen flow followed by desiccation in vacuum for 2 h. 30 nmol of the peptide maleimide lipid reaction mixture (containing 10 nmol Syntaxin lipopeptide and 20 nmol DOPE-maleimide lipid) in 50 mM HEPES, 140 mM KCl, 1 mM TCEP, and 3.5% OG was added to the dried lipids to make liposomes with Syntaxin lipopeptide. Alternatively, to make lipopeptide-free liposomes, 30 nmol DOPE-Maleimide lipid in 50 mM HEPES, 140 mM KCl, 1 mM TCEP, and 3.5% OG was added. OG-free buffer containing 50 mM HEPES, 140 mM KCl, and 1 mM TCEP was then added to reduce OG concentration to 1%. The mixture was resuspended by vortexing. Additional OG-free buffer was added to dilute OG to about 0.36% in final solution, below the critical micelle concentration. The solution was then dialyzed to remove OG and small molecules, similarly as previously described (39, 41), thereby forming SUVs.

### Bilayer Preparation and TIRF Microscopy.

The lipid bilayers were prepared by spontaneously bursting SUVs on the surface of the glass coverslip inside the channel of ibidi chips. Imaging was performed using a Nikon TIRF microscope, similarly to previous reports (22, 26).

### Counting Munc13 Copy Numbers.

We have previously described the procedure for counting the copy number of molecules in clusters of various sizes for determining the sizes of clusters in equilibrium experiments (14, 22, 26). Here is a brief description of the method.

Determination of the number of copies of Munc13 in each cluster is achieved through counting the number of fluorophores in the cluster.

For clusters at equilibrium, we gradually bleached the image frames using suitable laser power at different positions. The bleaching profiles of each particle (particle fluorescence intensity versus bleaching time) were plotted and, depending on the cluster size, two types of bleaching patterns were found. The bleaching profile displayed apparent discrete steps (14, 26) when the protein copy number was small. Then, the actual number of proteins and the bleaching step of a single fluorophore can be determined from counting the number and intensity of steps. The bleaching profile becomes smooth and can be fitted with an exponential decay function (14) when the copy number is large. The size of larger clusters can be calculated by dividing the initial fluorescence intensity of the cluster at the beginning of bleaching by bleaching step.

### Cocluster Size and Estimate of the PI(4,5)P_2_ Enrichment in the Coclusters.

We used ImageJ to isolate and quantify the coclusters dimensions and estimate PI(4,5)P_2_ densities. For each set of images containing the fluorescence intensities of PI(4,5)P_2_ and the lipopeptide, we first generated a mask that hid the pixels from the lipopeptide image that are not in coclusters. We then applied this mask to the PI(4,5)P_2_ image and set these pixels to zero. The resulting image was an image with the fluorescence intensities of the pixels that are in coclusters. We then determined the size of the connected regions using the “Find Particle” tool in ImageJ. The average size, 1.7 µm^2^, is provided in the text and the error bar is the SD. Because of the high density of PI(4,5)P_2_ in the coclusters, it is self-quenched. Hence the PI(4,5)P_2_ fluorescence intensity only provides a lower bound to the actual PI(4,5)P_2_ density. To circumvent this issue and estimate the actual PI(4,5)P_2_ density, we measured the total PI(4,5)P_2_ fluorescence over the whole image and compared it to that in experiments without lipopeptide. As expected, we found that this total fluorescence is larger without lipopeptide than with lipopeptide. Assuming a uniform enrichment of PI(4,5)P_2_, we distributed the fluorescence increase over the areas occupied by the coclusters and added it to the actually measured fluorescence. The resulting fluorescence indicates a threefold estimated mean enrichment in the coclusters compared to the uniform distribution without lipopeptide. Because of the optical resolution (~300 nm) we are unable to determine whether this enrichment is actually uniform or if there are denser nanodomains that form within the clusters.

### Determination of the Best Oligomer Prediction from Poisson Analysis.

To determine if a Poisson prediction of the cluster size distribution fits well with the experimental data, we use the following two parameters. First, for each oligomeric state *K-mer*, we use the inverse of the sum of the square of the difference between the predicted and observed probabilities:

[1] $$V=\frac{1}{\sum_{N=1}^{N_{\max}}{\left(P_{\mathrm{observed}}\left(N\right)-P_{\mathrm{Total}}\left(N\right)\right)}^{2}}.$$

Here we set *N_max_* = 10.

To ensure the results were solid, we used a second oligomerization test, *O*, represented by the inverse of the sum of the square of the relative error of the predicted probability multiplied by the observed probability:

[2] $$O=\frac{1}{\sum_{N=1}^{N_{\max}}{\left(\frac{P_{\mathrm{observed}}\left(N\right)-P_{\mathrm{Total}}\left(N\right)}{P_{\mathrm{observed}}\left(N\right)}\right)}^{2}P_{\mathrm{observed}}\left(N\right)}.$$

The term $P_{\mathrm{observed}}\left(N\right)$ is added to avoid that a large relative error on a rare event dominates the denominator.

Values of both the *V* and *O* parameters increase as the predicted distribution more closely matches the observed distribution.

### Vesicle Binding Modeling.

From the best Poisson distribution prediction considering each cluster as a mixture of oligomers of precise size and monomers, it is possible to determine the probability distribution of the number of oligomers, *n*, in each cluster of size *N*, *P_n_*(*N*). Then, assuming that *n* oligomers have a probability *P_n_* of binding a vesicle, the probability for a cluster of size *N* to bind a vesicle is $P_{\mathrm{bind}}\left(N\right)=\sum_{1}^{\infty }p_{n}P_{n}\left(N\right)$.

We performed this analysis on both wild-type trimers and Munc13 upright trimeric interface mutant hexamers. Under our experimental conditions, a single trimer had a 0.17 probability of capturing a vesicle. This probability increased to 0.55 with two trimers and reached 1.0 with three or more trimers. In contrast, a single hexamer had a vesicle capture probability of 0.8, while two or more hexamers consistently captured a vesicle.

### Cryo-EM Sample Preparation, Data Acquisition, and Data Processing.

For cryo-EM sample preparation, 75 µL of 7 mM lipids dissolved in 80:20 chloroform/methanol solution (33% DOPC, 60% DOPS, 6% PIP2, 1% Atto-465) was dried for 7 min under N_2_ gas and then placed under vacuum for 90 min. The dried lipids were resuspended in 75 µL of 125 mM HEPES 7.4, 600 mM KCl, 1 mM TCEP, and 1% Triton X-100 solution containing Munc18/Syntaxin and palmitoylated SNAP25 (pSNAP25) with a protein: lipid ratio of 1:800. The mix was incubated for 30 min at RT with gentle shaking (700 rpm vortex) followed by detergent removal by biobeads and then overnight dialysis against 4L of 125 mM HEPES 7.4, 600 mM KCl, 1 mM TCEP in cold room using a 7 kDa cutoff dialysis cassette (19). Next day, 4 µL of the dialyzed liposomes were supplemented with sucrose (Final concentration of 0.4 mM) and then air-dried on a MatTek dish for 30 min at RT. After 30 min, the lipid film was rehydrated with 10 µL of Milli-Q water and allowed to air-dry for a second time at RT for 30 min. Last, the dried lipid film was rehydrated in 20 µL suspension of vSUVs (63% DOPC, 15% DOPS, 20% Cholesterol, 2% Atto647-PE; Syt1:VAMP2:Syp = 22:60:30 copies outside/50 nm liposome, preincubated with 10 µM Complexin-1_26-83_, 200 nM Munc13C, and supplemented with 1 mM MgCl_2_) in 25 mM HEPES 7.4, 140 mM KCl, and 1 mM TCEP for 40 min at RT. After rehydration the mix was carefully resuspended and 3 µL was directly applied to a glow-discharged Lacey carbon supported copper grid (300 mesh). The grids were blotted for 5 s with a Whatman filter paper at 8 °C and 100% humidity and rapidly plunge frozen into liquid ethane cooled down by liquid nitrogen using a Mark IV Vitrobot.

The vitrified samples were imaged using a 200 kV Glacios Cryo-TEM microscope (Thermo Fisher Scientific) equipped with a K3 Summit direct electron detector (Gatan). Low and medium resolution maps were acquired for complete grid overview and to identify regions with thin ice suitable for cryo-ET imaging. Tomographic series were acquired using SerialEM (42) with a bidirectional tilt scheme starting from 0° with the following parameters: 13,500x nominal magnification resulting in a pixel size of 3.25 Å; tilt range ±51°; tilt increment 3°; 35 frames; total dose ∼80 e^−^/Å^2^.

The collected tilt movies were first subjected to motion correction using MOTIONCOR2 (43) and then assembled into drift-corrected stack files using *alignframes* from the IMOD software package (44). The aligned tilt series stacks were reconstructed by the Simultaneous Iterative Reconstruction Technique (SIRT) (45) using AreTomo software package (46) to produce non-CTF corrected 8x binned tomograms with a pixel size of 2.6 nm. The tomograms were denoised using Topaz (47) for further visualization and analysis. Pretrained denoising model unet-3d-20a was used for denoising.

### Cryo-EM Modeling and Visualization.

IMOD and University of California, San Francisco (UCSF) Chimera (48) were used to visualize the tomograms. Subtomograms (10 × 10 × 20 and 10 × 15 × 20 voxels for Fig. 5 *B* and *C* respectively) at 2.6 nm/voxel were extracted using IMOD from denoised tomograms and visualized in Chimera. The atomic model of Munc13-1 C_1_-C_2_B-MUN-C_2_C upright conformation (PDB 7T7X) (25) was manually placed into the surface rendering of the extracted 3D volumes of Munc13 trimeric assemblies using Chimera. During the docking process, the C_1_-C_2_B domains of Munc13-1 were placed proximally to the tGUV membrane and C_2_C was projected away from the membrane.

### Coarse-Grained Simulations.

First, a lipid bilayer was prepared using the Martini 3 force field (49). The lipid composition matched the experimental setup, except that the PI(4,5)P_2_ concentration was increased to 8% instead of the experimental 2% in order to accelerate the binding of Syntaxin lipopeptide to PI(4,5)P_2_ molecules. The system dimensions were set to (20 × 20 × 20) nm^3^. The lipid bilayer was generated using INSANE (50) in combination with Martini 3 Phosphoinositide Parameters (51) to accurately represent PI(4,5)P_2_ lipids. Two systems were created: a control membrane without lipopeptides, and another containing eight Syntaxin-1A lipopeptides, modeled as the Syntaxin-1A juxtamembrane peptide covalently linked to Maleimide-DOPE, following the experimental conjugation strategy. The Syntaxin-1A juxtamembrane peptide was first modeled using AlphaFold2 (52) to obtain its atomistic structure, which was subsequently converted into a coarse-grained representation and topology using Martinize2 (53) and finally covalently attached to a coarse-grained DOPE lipid through the Maleimide-specific linker (*SI Appendix*, Fig. S1). The lipopeptides were placed in the same leaflet as PI(4,5)P_2_ to allow direct interaction. Both systems (with and without lipopeptides) were then solvated, and electrical neutrality was achieved by adding counterions.

All simulations were carried out using the GROMACS 2023 software package (54). Energy minimization was performed using the steepest descent method, followed by four equilibration phases. Throughout equilibration, the temperature was maintained at 310 K using the Berendsen thermostat (55) with a time constant of 1 ps, applied separately to the solvent, membrane, and peptide groups (when present). In the first equilibration step, pressure was controlled semi-isotropically using the Berendsen barostat with a time constant of 10 ps and a reference pressure of 1 bar in both x–y and z directions. During the second and third equilibration phases, the Berendsen thermostat was retained, but the pressure coupling time constant was reduced to 3 ps to facilitate relaxation, while keeping reference pressures and compressibility values unchanged. In the fourth equilibration step, the thermostat was switched to velocity rescaling (V-rescale) (56) with the same temperature-coupling groups, and pressure was maintained at 1 atm using the C-rescale barostat (57) with a coupling constant of 4 ps. After equilibration, the system was subjected to a 20-μs production run using a 20-fs integration time step, with temperature controlled via the V-rescale thermostat and pressure maintained semi-isotropically using the C-rescale barostat under the same conditions as during the final equilibration stage. All input files and the corresponding MD trajectory are available at Zenodo: 10.5281/zenodo.17873155.

We focused on two types of interactions: 1) interactions between PI(4,5)P_2_ lipids themselves, related to cluster formation, and 2) interactions between the peptides and PI(4,5)P_2_ lipids. The first type of interaction refers to the number of contacts, that is, the number of PI(4,5)P_2_ headgroups located within 1 nm of a given PI(4,5)P_2_ lipid (*SI Appendix*, Fig. S2*A*), evaluated in both the control system (PI(4,5)P_2_ cluster (No peptide)), where PI(4,5)P_2_ forms clusters in the absence of peptide, and in the peptide-containing system. In the latter, we distinguish between clusters that are in contact with at least one peptide at a distance of 1 nm or less (PI(4,5)P_2_ cluster near peptide), and clusters that are farther away from any peptide (PI(4,5)P_2_ cluster far from peptide). We then calculated the average cluster size for this type of interaction. To assess statistical significance, we performed pairwise comparisons using Dunn’s test, following confirmation that the data did not follow a normal distribution according to the Anderson–Darling test.

The second type of interaction is defined by the number of PI(4,5)P_2_ headgroups located within 1 nm of a peptide (*SI Appendix*, Fig. S2*B*). These interactions were quantified by analyzing the simulation trajectory. VMD (58) was used to display the membrane system containing peptides.

To further resolve the molecular determinants of PI(4,5)P_2_ binding, we also quantified residue-level interactions along the trajectory. For each residue of the Syntaxin-1A peptide, we calculated the mean number of PI(4,5)P_2_ headgroups located within 1 nm, generating a per-residue contact profile (Fig. 1*C*) that reveals which residues contribute most strongly to PI(4,5)P_2_ binding across the simulation.

In order to calculate the surface density of PI(4,5)P_2_ molecules over time (Fig. 1*D*) in regions of the membrane with and without PIP_2_-lipopeptide clusters, we counted the number of PI(4,5)P_2_ molecules in contact with peptides and those that are not. Additionally, we measured the membrane area in contact with the peptides and the area without contact. In general, dividing the number of PI(4,5)P_2_ molecules by the corresponding membrane area yields the surface density, both for PI(4,5)P_2_ near the peptides (PIP_2_ cluster near peptide) and for those located farther away (PIP_2_ cluster far from peptide).

## Supplementary Material

Appendix 01 (PDF)

Movie S1. Movie of the representative tomogram from Figure 5A, reconstructed at bin 8 (2.6 nm/pixel) and denoised with Topaz. Yellow labels indicate a Munc13C monomer protruding from the membrane (‘Monomer’) and Munc13 trimeric clusters on the GUV surface (‘Trimers’).

Movie S2. Movie of the isolated Munc13 trimer subtomogram from Figure 5B. The atomic model of the C_1_-C_2_B-MUN-C_2_C fragment in the upright conformation is depicted in orange (PDB 7T7X, (1)). The scale bar is 5 nm.

Movie S3. Movie of the subtomogram of two clustered Mucn13 trimer units from Figure 5C. The atomic model of the C_1_-C_2_B-MUN-C_2_C fragment in the upright conformation is depicted in orange (PDB 7T7X, (1)). The scale bar is 5 nm.

## Data Availability

Study data are included in the article and/or supporting information.
