# Supplementary material for: Synaptic transmission: Munc13 assembles onto PI(4,5)P2-rich domains into trimers that cooperate to capture vesicles
Source: Proc Natl Acad Sci U S A. 2026 Feb 11;123(7):e2523347123. doi: 10.1073/pnas.2523347123 (PMC12912961; doi:10.1073/pnas.2523347123)
Supplement: Supplementary file 1 — Appendix 01 (PDF) [file pnas.2523347123.sapp.pdf]

PNAS

Supporting Information for

Synaptic Transmission: Munc13 assembles onto PI(4,5)P_2-rich domains into trimers that cooperate to capture vesicles

Feng Li, Abhijith Radhakrishnan, Sudhanshu Gautam, Gabriel Diaz, Venkat Kalyana Sundaram, Jeff Coleman, Hong Zheng, Kirill Grushin, Matthieu Chavent, James E. Rothman, and Frederic Pincet

Corresponding authors: James E. Rothman, Frederic Pincet
Email: james.rothman@yale.edu, frederic.pincet@ens.fr

This PDF file includes:

Figures S1 to S8
Legends of Supporting Videos S1 to S3

Fig. S1.

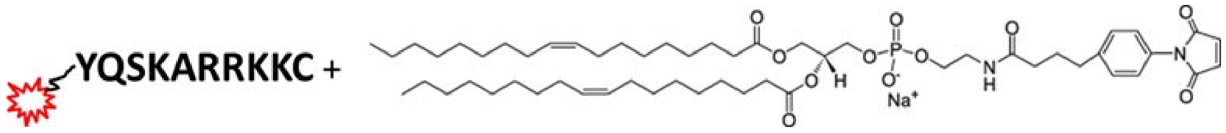

Supporting Figure S1. Illustration of the reaction between the C-terminal Cysteine on the Syntaxin-1A juxtamembrane peptide with Maleimide-DOPE lipid to lapidated peptide. Different variations of conjugations were made on the N-terminus of the peptide, including: (1) No fluorescent molecule was conjugated; or (2) fluorescent molecule such as fluorescein, Sulforhodamin10, or CyLyte Fluor5, was conjugated on the N-terminus of the peptide.

Fig. S2.

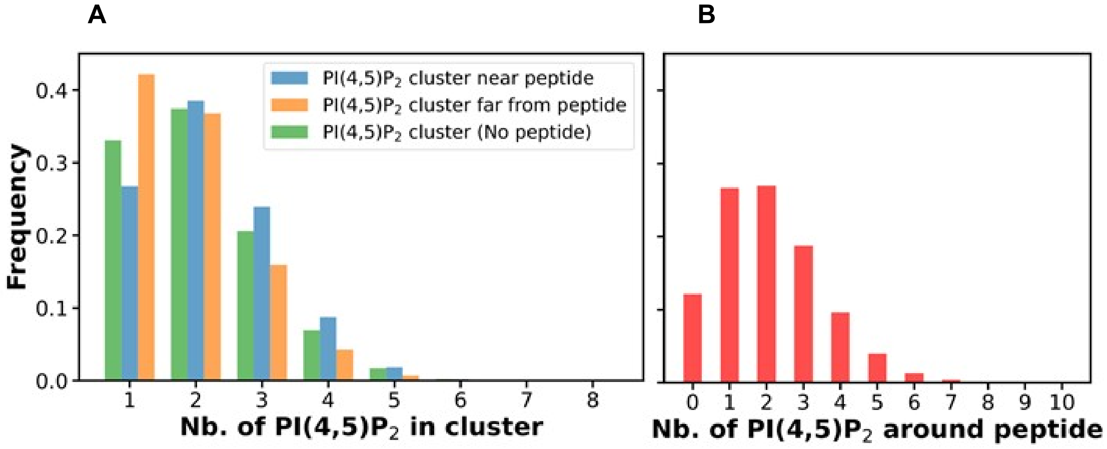

Supporting Figure S2. $PI(4,5)P_2$ **number of contacts. (A)** Distribution of the number of $PI(4,5)P_2$ molecules forming clusters under three conditions: a reference system in the absence of lipopeptide ($PI(4,5)P_2$ cluster with no peptide, green bars), and a system with lipopeptide, in which $PI(4,5)P_2$ molecules are classified as either near the peptide ($PI(4,5)P_2$ near peptide, blue bars) or far from it ($PI(4,5)P_2$ far from peptide, orange bars). The average cluster sizes for the No peptide, $PI(4,5)P_2$ near peptide, and $PI(4,5)P_2$ far from peptide conditions were 2.11, 2.21, and 1.84 $PI(4,5)P_2$ molecules per cluster, respectively. Differences between the three conditions were statistically significant ($p < 0.001$). (**B)** Distribution of the number of $PI(4,5)P_2$ molecules around a peptide within 1 nm, with an average of 2.07 molecules per peptide.

Fig. S3.

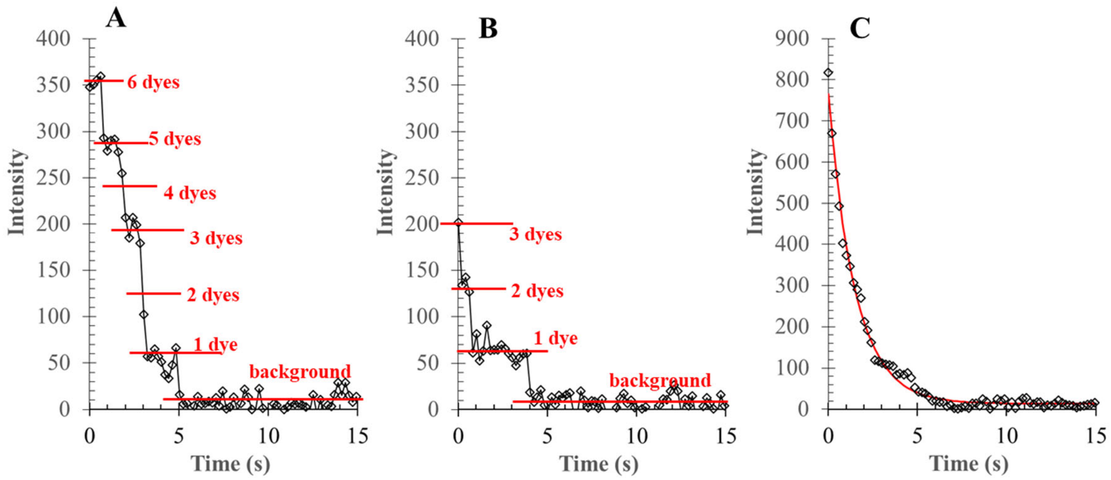

Supporting Figure S3. Example of cluster bleaching traces. (A) and (B) are examples of step-bleaching traces (Munc13 copy number below 7) for Munc13 clusters containing respectively 6 and 3 Munc13 copies and (C) is an example of cluster containing more than 6 Munc13 copies. In that case the plateaus observed in A and B are too short because of the large copy number. The bleaching curve appears smooth and can be fitted by $I(t) = I_0 e^{-t/t_0} + B$. Here I_0 = 752. With our setting, the step corresponding to one dye is on average 62. Hence, there are approximately a 12 Munc13 copy number in this example.

Fig. S4.

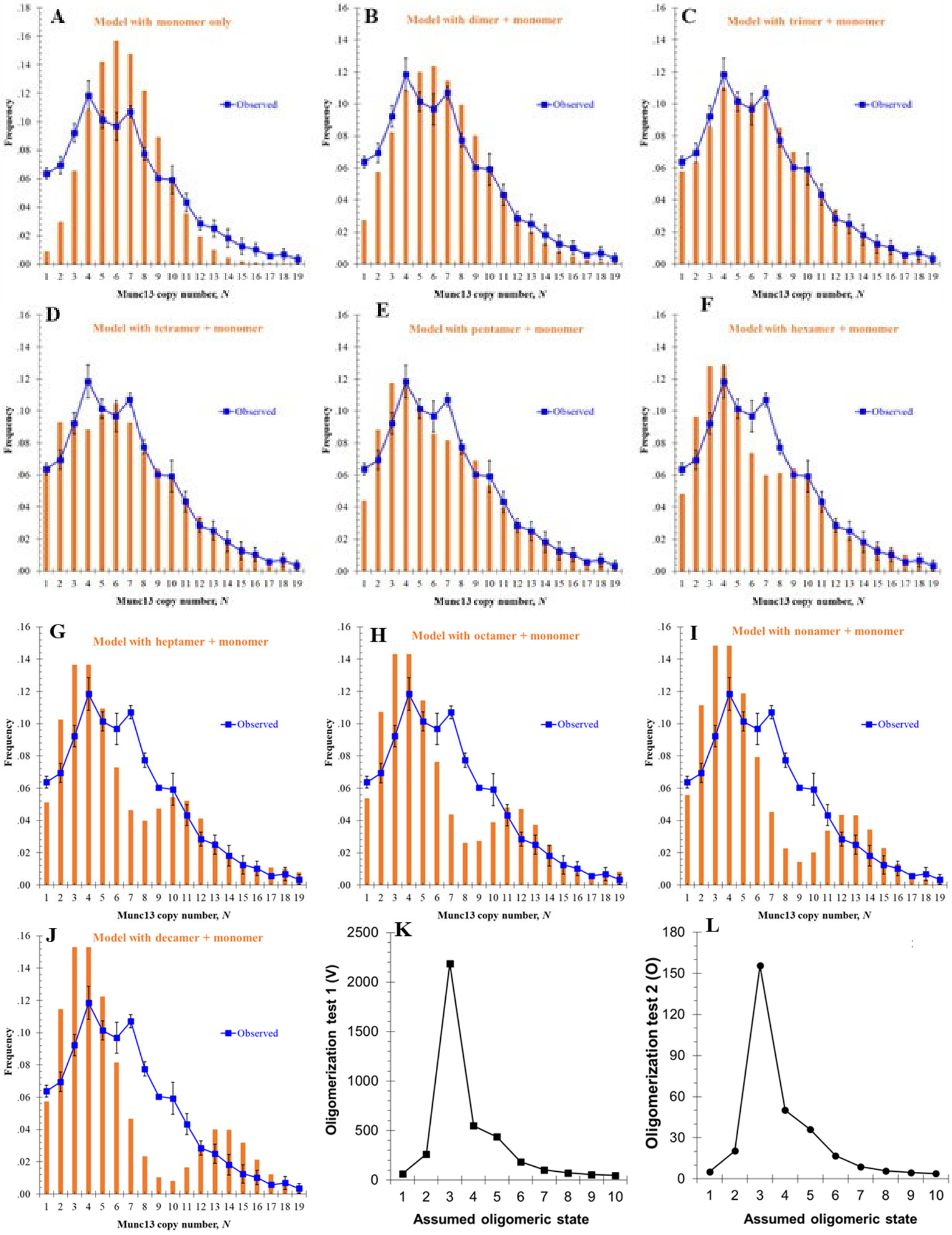

Supporting Figure S4. Poisson distribution modeling of oligomers in wildtype Munc13 clusters on PI(4,5)P_2 domains. (**A**) Single Poisson distribution, assuming all wildtype Munc13 in monomer state; (**B**) through (**J**) Dual Poisson distribution (monomer + uniform size oligomer), 2-step process: monomer with dimer (**B**), monomer with trimer (**C**), monomer with tetramer (**D**), monomer with pentamer (**E**), monomer with hexamer (**F**), monomer with heptamer (**G**), monomer with octamer (**H**), monomer with nonamer (**I**), and monomer with decamer (**J**). The orange bars represent predicted distribution of the copy number of Munc13 molecules in the clusters, and the blue lines are experimental data. (**K**) Quantification of the inverse of the sum of the square of the difference between measurement and predictions, V, described in Eq. 1, which shows the inverse of the least square of the absolute variation between the measured and predicted size distribution for each assumed oligomeric state of wildtype Munc13. (**L**) The *O* value of the Poisson modelling, described in Eq. 2, as a function of the assumed oligomeric state of wildtype Munc13.

Fig. S5.

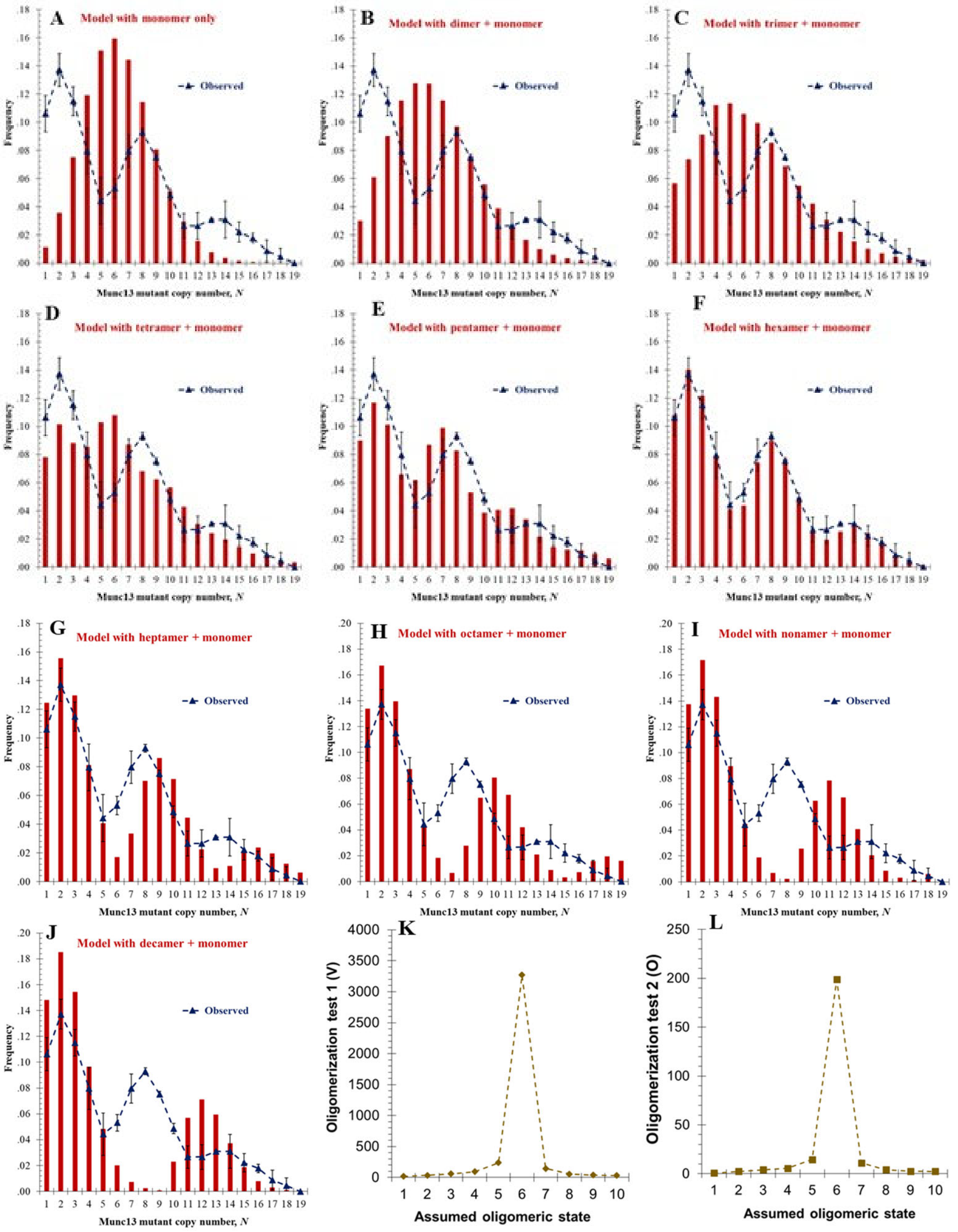

Supporting Figure S5. Poisson distribution modeling of oligomers in Munc13 upright trimeric interface mutant clusters on PI(4,5)P_2 domains. (**A**) Single Poisson distribution, assuming all Munc13 mutant in monomer state; (**B**) through (**J**) Dual Poisson distribution (monomer + uniform size oligomer), 2-step process: monomer with dimer (**B**), monomer with trimer (**C**), monomer with tetramer (**D**), monomer with pentamer (**E**), monomer with hexamer (**F**), monomer with heptamer (**G**), monomer with octamer (**H**), monomer with nonamer (**I**), and monomer with decamer (**J**). The dark red bars represent predicted distribution of the copy number of Munc13 molecules in the clusters, and the dark blue dashed lines are experimental data. (**K**) Quantification of the inverse of the sum of the square of the absolute difference between measurement and predictions, V, described in Eq. 1, which shows the inverse of the least square of the absolute variation between the measured and predicted size distribution for each assumed oligomeric state of Munc13 trimeric interface murant. (**L**) The *O* value of the Poisson modelling, described in Eq. 2, as a function of the assumed oligomeric state of Munc13 interface mutant.

Fig. S6.

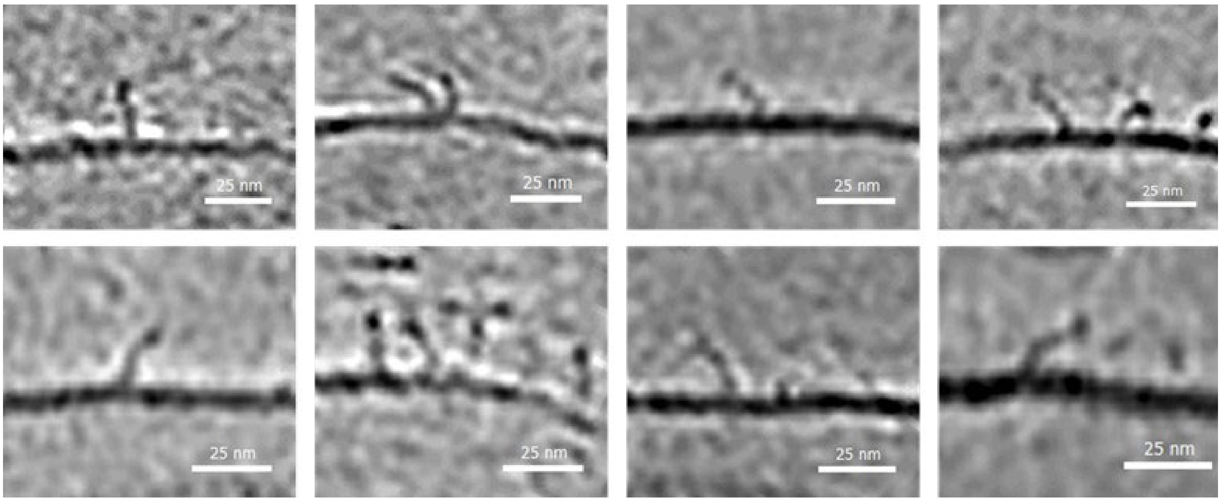

Supporting Figure S6: Munc13C monomers on negatively charged GUVs visualized by cryo-ET. Tomographic XY slices show rod-shaped Munc13C molecules on the GUV surface, appearing flexible and adopting multiple conformations.

Fig. S7.

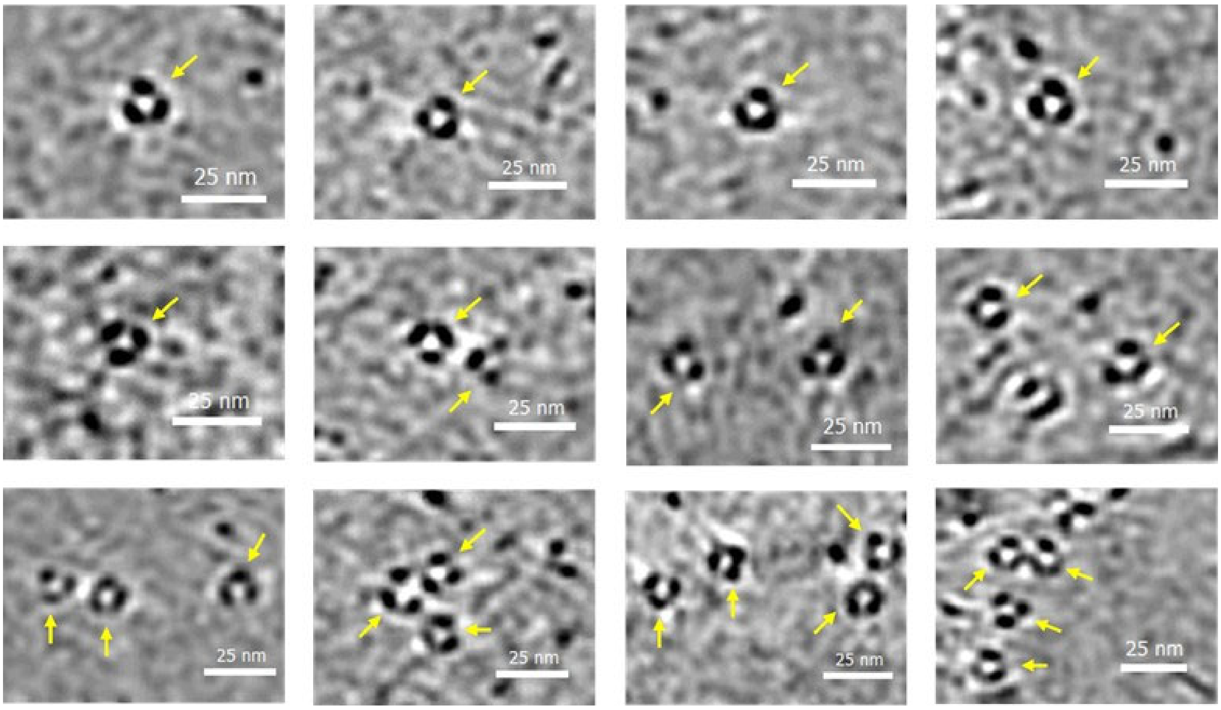

Supporting Figure S7: Isolated and clustered Munc13C trimers on negatively charged GUVs. Tomographic XY slices show isolated and clustered Munc13C trimers (yellow arrows) on the GUV surface. We did not observe trimers of Munc13C in the side-view orientation; therefore, all images shown are in top-view orientation.

Fig. S8.

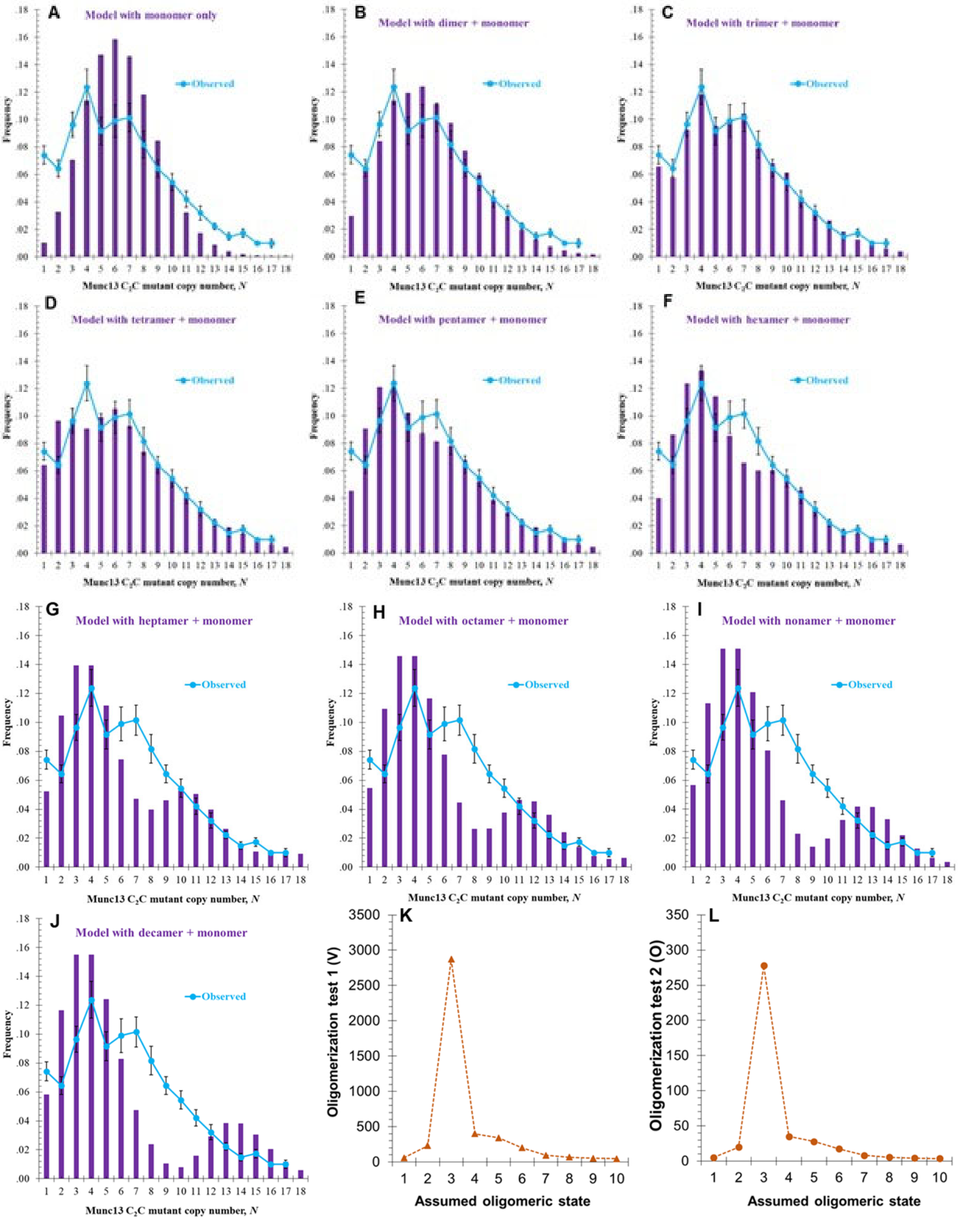

Supporting Figure S8. Poisson distribution modeling of oligomers in Munc13 C$_2$C mutant clusters on PI(4,5)P$_2$ domains. (**A**) Single Poisson distribution, assuming all Munc13 C$_2$C mutant in monomer state; (**B**) through (**J**) Dual Poisson distribution (monomer + uniform size oligomer), 2-step process: monomer with dimer (**B**), monomer with trimer (**C**), monomer with tetramer (**D**), monomer with pentamer (**E**), monomer with hexamer (**F**), monomer with heptamer (**G**), monomer with octamer (**H**), monomer with nonamer (**I**), and monomer with decamer (**J**). The purple bars represent predicted distribution of the copy number of Munc13 C$_2$C mutant molecules in the clusters, and the cyan solid lines are experimental data. (**K**) Quantification of the inverse of the sum of the square of the absolute difference between measurement and predictions, V, described in Eq.1, which shows the inverse of the least square of the absolute variation between the measured and predicted size distribution for each assumed oligomeric state of Munc13 C$_2$C mutant. (**L**) The *O* value of the Poisson modelling, described in Eq. 2, as a function of the assumed oligomeric state of Munc13 C$_2$C mutant.

Supporting Video Legends

Video S1: Movie of the representative tomogram from Figure 5A, reconstructed at bin 8 (2.6 nm/pixel) and denoised with Topaz. Yellow labels indicate a Munc13C monomer protruding from the membrane (‘Monomer’) and Munc13 trimeric clusters on the GUV surface (‘Trimers’).
Video S2: Movie of the isolated Munc13 trimer subtomogram from Figure 5B. The atomic model of the C_1-C_2B-MUN-C_2C fragment in the upright conformation is depicted in orange (PDB 7T7X, (1)). The scale bar is 5 nm.
Video S3: Movie of the subtomogram of two clustered Mucn13 trimer units from Figure 5C. The atomic model of the C_1-C_2B-MUN-C_2C fragment in the upright conformation is depicted in orange (PDB 7T7X, (1)). The scale bar is 5 nm.
